# Differential Effect of Non-Steroidal Anti-Inflammatory Drugs Aspirin and Naproxen against *TMPRSS2-ERG* (Fusion)-Driven and Non-Fusion-Driven Prostate Cancer

**DOI:** 10.3390/cancers15205054

**Published:** 2023-10-19

**Authors:** Komal Raina, Kushal Kandhari, Rama Kant, Ram Raj Prasad, Neha Mishra, Akhilendra K. Maurya, Jennifer T. Fox, Shizuko Sei, Robert H. Shoemaker, Maarten C. Bosland, Paul Maroni, Chapla Agarwal, Rajesh Agarwal

**Affiliations:** 1 Department of Pharmaceutical Sciences, Skaggs School of Pharmacy and Pharmaceutical Sciences, University of Colorado Anschutz Medical Campus, Aurora, CO 80045, USA; kushal.kandhari@cuanschutz.edu (K.K.); rama.kant@cuanschutz.edu (R.K.); ram.prasad@cuanschutz.edu (R.R.P.); neha.mishra@cuanschutz.edu (N.M.); akhilendrakumar@bhu.ac.in (A.K.M.); chapla.agarwal@cuanschutz.edu (C.A.); 2 Department of Pharmaceutical Sciences, South Dakota State University, Brookings, SD 57007, USA; 3 Chemopreventive Agent Development Research Group, Division of Cancer Prevention, National Cancer Institute, NIH, Bethesda, MD 20892, USA; jennifer.fox@nih.gov (J.T.F.); seis@mail.nih.gov (S.S.); shoemakr@mail.nih.gov (R.H.S.); 4 Department of Pathology, College of Medicine, University of Illinois at Chicago, Chicago, IL 60612, USA; boslandm@uic.edu; 5 Department of Surgery, Division of Urology, University of Colorado Anschutz Medical Campus, Aurora, CO 80045, USA; paul.maroni@cuanschutz.edu; 6 University of Colorado Cancer Center, University of Colorado Anschutz Medical Campus, Aurora, CO 80045, USA

**Keywords:** prostate cancer, *TMPRSS2-ERG* fusion, Hi-Myc mice, aspirin, naproxen, NSAID

## Abstract

**Simple Summary:**

Disparity in clinical outcome data due to the biological heterogeneity of prostate cancer (PCa) has drawn attention to approaches that stratify homogeneous subsets of patients. In recent years, PCa fusion genes (specifically *TMPRSS2-ERG* fusion) have been identified as oncogenic drivers with the potential for patient stratification and as targets for effective prevention/intervention strategies in drug efficacy trials. In the present study, employing relevant *TMPRSS2-ERG* (fusion)-driven and non-*TMPRSS2-ERG*-driven mouse models of PCa, we report the potential usefulness of the non-steroidal anti-inflammatory drugs (NSAIDs) aspirin and naproxen specifically against *TMPSS2-ERG* fusion-driven prostate tumorigenesis. These findings are consistent with the clinical observations and warrant further investigation of the molecular mechanisms and utility of NSAID interventions for precision cancer prevention.

**Abstract:**

The consumption of the non-steroidal anti-inflammatory drug (NSAID) aspirin is associated with a significant reduction in the risk of developing *TMPRSS2-ERG* (fusion)-positive prostate cancer (PCa) compared to fusion-negative PCa in population-based case–control studies; however, no extensive preclinical studies have been conducted to investigate and confirm these protective benefits. Thus, the focus of this study was to determine the potential usefulness of aspirin and another NSAID, naproxen, in PCa prevention, employing preclinical models of both *TMPRSS2-ERG* (fusion)-driven (with conditional deletion of *Pten*) and non-*TMPRSS2-ERG*-driven (Hi-Myc^+/−^ mice) PCa. Male mice (*n* = 25 mice/group) were fed aspirin- (700 and 1400 ppm) and naproxen- (200 and 400 ppm) supplemented diets from (a) 6 weeks until 32 weeks of Hi-Myc^+/−^ mice age; and (b) 1 week until 20 weeks post-Cre induction in the fusion model. In all NSAID-fed groups, compared to no-drug controls, there was a significant decrease in higher-grade adenocarcinoma incidence in the *TMPRSS2-ERG* (fusion)-driven PCa model. Notably, there were no moderately differentiated (MD) adenocarcinomas in the dorsolateral prostate of naproxen groups, and its incidence also decreased by ~79–91% in the aspirin cohorts. In contrast, NSAIDs showed little protective effect against prostate tumorigenesis in Hi-Myc^+/−^ mice, suggesting that NSAIDs exert a specific protective effect against *TMPRSS2-ERG* (fusion)-driven PCa.

## 1. Introduction

Population-based case–control studies have highlighted the potential benefits of the non-steroidal anti-inflammatory drug (NSAID) aspirin intake against the risk of developing prostate cancer (PCa) [1,2,3]. Notably, a recent small population-based case–control study identified an association between aspirin use and a reduction in the transmembrane protease serine 2-Ets (erythroblastosis virus E26)-related gene [*TMPRSS2-ERG*] fusion-positive PCa [2]. Among men reporting current aspirin use, there was a ~37% reduction in the risk of developing *TMPRSS2-ERG-*positive PCa, with a stronger risk reduction observed with longer durations of use [2]. No association with aspirin use was seen in *TMPRSS2-ERG-*negative PCa [2]. However, no extensive preclinical efficacy studies have been conducted to confirm these protective benefits or to investigate the mechanistic basis. Thus, it is imperative to carry out efficacy studies in *TMPRSS2-ERG-*driven and non-*TMPRSS2-ERG-*driven PCa preclinical animal models and examine the antitumor efficacy of aspirin against PCa development and progression (either dependent or independent of *TMPRSS2-ERG* fusion status) and further to assess whether NSAIDs other than aspirin can also confer similar protective effects against PCa.

The mechanisms that may underlie the preventive activity of aspirin and other NSAIDs against cancer are complex [4,5]. However, their anti-inflammatory activity, including inhibition of cyclooxygenases 1 and 2 (COX-1, -2), resulting in a reduction of several prostaglandins (PGs) as well as thromboxane A, are recognized as potential anti-cancer mechanisms [4,5]. This may be particularly applicable to PCa, in which inflammation, although not necessarily a trigger for tumorigenesis, may contribute to tumor progression mediated by the overexpression of COX-2, particularly in higher-stage PCa [6,7], leading to the overproduction of the inflammation mediators such as PGs, and thromboxane A [8]. Of note, COX-2, unlike the constitutively active COX-1 enzyme, is inducible by cytokines and growth factors [7]. Notably, aspirin intake has been associated with gastrointestinal complications arising from COX-1 inhibition, and various NSAIDs, especially the COX-2 selective inhibitors, have been implicated in human cardiovascular (CV) risk [4,5,9]. Given that a comparative systematic review identified that in the non-aspirin NSAID users, the lowest CV risks were associated with naproxen, a non-selective NSAID [9], we decided to evaluate the chemopreventive efficacy of aspirin as well as naproxen against prostate tumorigenesis in relevant preclinical models.

The overall objective of the present study was to determine the potential clinical usefulness of aspirin and naproxen for PCa prevention employing preclinical models of *TMPRSS2-ERG* fusion- and *Pten* loss-driven (given that *Pten* loss together with the *TMPRSS2-ERG* fusion initiate neoplastic events) [10] as well as non-*TMPRSS2-ERG-*driven PCa tumorigenesis. Furthermore, extensive studies were performed using these models to delineate the mechanisms that may be involved in the efficacy of these NSAIDs, and possible intermediate biomarkers potentially involved in mediating the protective effects of aspirin and naproxen were critically examined.

## 2. Materials and Methods

### 2.1. Animals

Homozygous male and female *TMPRSS2-ERG. Pten^flox/flox^* mice were crossed to generate *TMPRSS2-ERG. Pten^flox/flox^* mice (breeding pairs were a kind gift from Dr. Yu Chen, Memorial Sloan-Kettering Cancer Center, New York) [11,12]. At 8 weeks of age, the male offsprings were administered tamoxifen for Cre-induction as described previously [12].

Hi-Myc mice [Hi-Myc^+/−^ (ARR2PB-Myc, FVB/N)] were used as a non-*TMPRSS2-ERG* fusion-driven PCa model. This mouse model is a spontaneous model of PCa, where tumorigenesis is Myc-driven (androgen-dependent, prostate-specific ARR2/probasin-cMyc transgene expression); this model is extensively used in pre-clinical anti-PCa efficacy studies [13,14,15]. Cryopreserved Hi-Myc mouse embryos were acquired from NCI and re-derived. Male Hi-Myc mice were generated by breeding female Hi-Myc^+/−^ with wild-type FVB male mice (Charles River Labs., Wilmington, MA, USA) as described previously [12]. Animal care and treatments were in accordance with institutional guidelines (University of Colorado Denver Animal Care and Use Committee) under approved protocol #B-57915(02)1E (approved on 9 August 2016). Mice of the relevant genotype were randomized into different groups (as and when the litters were available).

### 2.2. Animal Diets

Throughout the study, all mice were fed a powdered semipurified diet (gamma irradiated modified AIN-76A: Envigo# TD 94096 diet). In the NSAID diet-fed cohorts, one week after Cre-induction, the control TD 94096 powder diet was switched to a TD 94096 diet supplemented with different doses (human equivalent doses) of aspirin or naproxen [16]. Treatment drugs were aspirin (Sigma Aldrich, St. Louis, MO, USA: cat# A2093) and naproxen (TCI Chemicals, Portland, OR, USA: cat# M1021). Aspirin 700 ppm and 1400 ppm doses were prepared by mixing 0.7 g or 1.4 g of aspirin in 1 kg of powder TD 94096 diet, respectively. Naproxen 200 ppm and 400 ppm doses were prepared by mixing 0.2 g or 0.4 g of naproxen in 1 kg of the powder diet. The powder was mixed uniformly using the geometric dilution approach and tumble blending. Since the animal facility was specifically pathogen-free, all drug-supplemented diets were prepared/mixed under aseptic conditions; supplemented diets were prepared fresh on the same day of utilization and feeding bowls were replenished daily with fresh powder-control or NSAID-supplemented diets.

In the *TMPRSS2-ERG. Pten^flox/flox^* efficacy study, 1 week post-Cre-induction (tamoxifen-induced at 8 weeks of age), mice were initiated on an NSAID-supplemented powder diet until the end of the study (20 weeks post-Cre-induction or 28 weeks of age). In the Hi-Myc^+/−^ efficacy study, the NSAID-supplemented diets were fed from 6 weeks of age until the study endpoint at 32 weeks of age. Animals were permitted free access to food and drinking water. Animal body weights were recorded weekly, and the animals were monitored daily for general health. As overall controls, age-matched non-Cre induced *TMPRSS2-ERG. Pten^flox/flox^* or wild-type (FVB) mice fed either a control diet or NSAID-supplemented diets (as per the above protocol) were also included in the study and sacrificed at the study endpoint. The group size was 25 mice for the control and NSAID-supplemented groups of the PCa models and 7 mice for the non-PCa controls. The efficacy study design with NSAIDs in the *TMPRSS2-ERG*-driven and non-*TMPRSS2-ERG*-driven (Hi-Myc^+/−^) PCa models is detailed in Supplementary Table S1.

### 2.3. Euthanasia and necropsy

Before necropsy, mice were weighed and then humanely euthanized using CO_2_ asphyxiation followed by exsanguination. The lower urogenital tract (LUT), including the bladder, seminal vesicles, and prostate, were removed en bloc, and the LUT wet weight was recorded (Supplementary Figure S1). Distinct sections of the prostate, including the anterior prostate (AP), ventral prostate (VP), and dorsolateral prostate (DLP) lobes, were carefully extracted and micro-dissected whenever feasible [when the tumor obscured the boundaries of lobes, they were taken as such (without micro-dissection)]. The entire prostate was either formalin-fixed or in some cases dissected in the middle and one portion was formalin-fixed and the other was snap-frozen and stored at –80 °C (for molecular analysis in future studies).

### 2.4. Histopathological and Immunohistochemical Evaluation of Prostatic Tissues

Multiple tissue sections were made from each prostatic tissue/tumor paraffin block. Serial tissue sections (5 µm thick), after every 5 such sections of paraffin-embedded tissues (three slides per tissue) were stained with hematoxylin and eosin (H&E) for histopathological evaluation. Stained sections were then examined under a microscope, and categorized according to the Bar Harbor classification of mouse prostate pathology into prostatic hyperplasia, prostatic intraepithelial neoplasia (PIN) lesions, microinvasive carcinoma, and well (WD)/moderately (MD)/poorly differentiated (PD) adenocarcinoma [17,18,19]. The maximum histological score for the prostate lobe was used to calculate a mean tumor grade for the group. Blinded analysis for histopathological data collection was performed only after an initial review of the possible pathology in these prostate tissues.

For immunohistochemistry (IHC), after deparaffinization, tissue sections were subjected to staining using specific primary/secondary antibodies (based on streptavidin-biotin system), followed by 3,3′-diaminobenzidine (DAB) staining, as per the guidelines provided by the manufacturer, and following previously established protocols [18,20]. Primary antibodies used were from Abcam, Waltham, MA, USA; [PCNA (#ab29), ERG (#ab92513), CK5 (3ab52635)], NFκB (#ab16502)]; and Santa Cruz Biotechnology Inc., Dallas, TX, USA; [androgen receptor (#sc816), c-Myc (#sc-40), prostein (#sc393069), PECAM-1/CD-31 (#sc376764), COX-2 (#sc-1747), COX-1 (#sc-1752), vimentin (V9 #sc-6260), CK8 (#sc-8020); Cell Signaling Technology, Danvers, MA, USA; E-cadherin (#3195), cleaved-caspase 3 (#9661)]. Biotinylated secondary antibodies used were from Dako/Agilent, Santa Clara, CA, USA; [rabbit anti-mouse (#E0464), rabbit anti-rat (#E0468), and goat anti-rabbit (#E0432)]; and Santa Cruz Biotechnology Inc., Dallas, TX, USA; [rabbit anti-goat #sc-2774]. Fluorescent tagged-secondary antibodies used were from Invitrogen, Waltham, MA, USA; [goat anti-mouse-Alexa Fluor 647 (#A21236), goat anti-rabbit-Alexa Flour 488 (#A11008)]. Since the most aggressive pathologies were observed in the DLP in both models, the IHC analysis was mostly conducted on these tissues [12].

### 2.5. Statistical and Microscopic Analyses

Data were analyzed using Sigma Stat/Graph Pad Prism version 8.0 Software. Two-sided *p* values less than 0.05 were considered significant. Fisher’s exact test was applied to compare the incidence of PIN and adenocarcinoma lesions. Unpaired two-tailed Student’s *t*-test or ANOVA was utilized for other data sets. Detailed statistical information for these assessments is provided in Supplementary Tables S2 and S3. Regarding the IHC studies, quantifying positive cells involved counting brown-stained cells within the total cell count, across 5–8 randomly selected fields at a magnification of ×400. This quantification was then expressed as a percentage of positive cells. The level of immunoreactivity, as indicated by the intensity of brown staining, was semiquantitatively graded as 0 (no staining), +1 (weak), +2 (moderate), +3 (strong), and +4 (very strong); slight variations in relative intensity were considered depending upon the specific molecular marker being examined. This grading system was slightly adapted to accommodate different types of staining (membrane, cytoplasmic, peri-nuclear, nuclear); further details can be found in the figure legends where the data are presented.

H-Score analysis for prostein expression was calculated as [% proportion area of the prostate (positive for prostein expression) × immunoreactivity score (prostein perinuclear intensity)]. For H-score calculations, the % proportion area was given arbitrary scores (<1% = 0, ≤2–10% = 1, ≤11–25% = 2, ≤26–50% = 3, ≤51–100% = 4). Variations among the groups were ascertained through either ANOVA or unpaired *t*-tests, depending on relevance, followed by Tukey’s test for conducting multiple comparisons. Mean values accompanied by their corresponding standard error of the mean (SEM) are presented to represent quantitative data. Microscopic evaluations were performed using a Zeiss Axioscope 2 microscope (Carl Zeiss, Jena, Germany). Photomicrographs were captured using an AxioCam MrC5 camera (Carl Zeiss, Jena, Germany) as shared in relevant figures and Supplementary Figure S2. Comparative data of IHC analysis in age-matched non-Cre induced *TMPRSS2-ERG. Pten*^flox/flox^ or wild-type (FVB) control mice fed either a control diet or NSAID-supplemented diets are shown in Supplementary Figures S3–S6.

## 3. Results

### 3.1. Effects of Aspirin and Naproxen Intervention on Cancer Progression in Different Prostate Lobes of TMPRSS2-ERG Fusion-Driven and Non-Fusion-Driven Hi-Myc^+/-^ PCa Models

No significant difference in body weight gain (LUT weights subtracted) between the efficacy study mice on control diets and NSAID-supplemented diets was observed. The overall control groups (no-TAM and WT-FVB mice), either fed with control diet or NSAID-supplemented diets, showed significantly lower LUT weights compared to their respective positive controls [(+TAM) and Hi-Myc^+/−^ mice]. There was no difference in LUT weights of *TMPRSS2-ERG Pten^flox/flox^* (+TAM) mice fed with control diet and NSAID-supplemented diets (Supplementary Figure S1). On the other hand, while the LUT weights of the Hi-Myc^+/−^ control mice were similar to mice fed with naproxen (200 and 400 ppm) and aspirin 700 ppm diets, a slight increase (~1 fold, *p* ≤ 0.05) in the LUT weight of aspirin 1400 ppm-fed mice was observed compared to Hi-Myc^+/−^ controls (Supplementary Figure S1).

**Figure 1:**
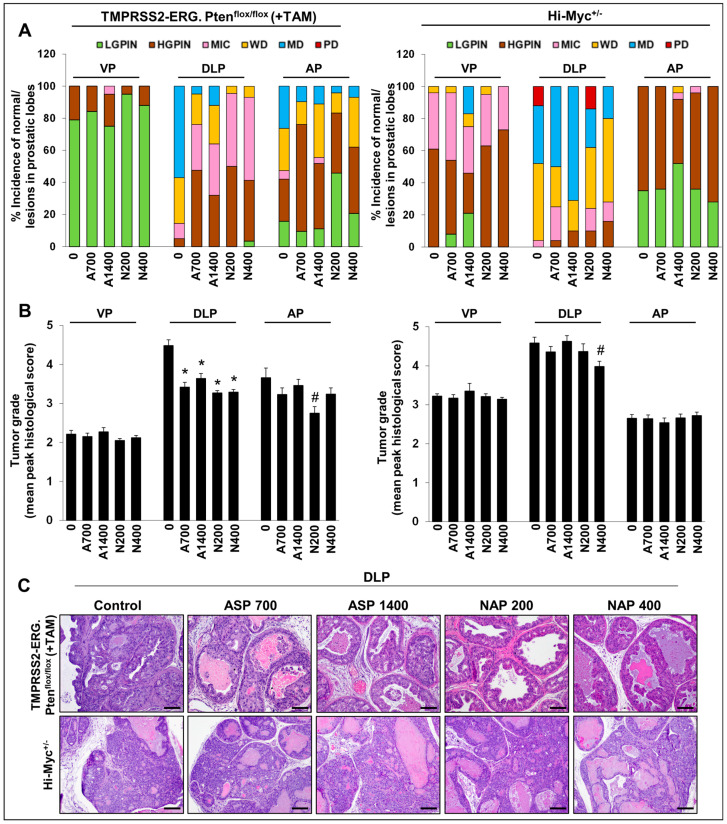
Effects of aspirin and naproxen interventions on cancer progression in different prostate lobes of *TMPRSS2-ERG* fusion-driven and non-fusion-driven Hi-Myc^+/−^ PCa models. NSAID effects on (**A**) % incidence of pre-/neoplastic and adenocarcinoma lesions, (**B**) tumor grades of histopathological lesions in different prostate lobes of *TMPRSS2-ERG. Pten^flox/flox^* (+TAM) (**left-panel**), and Hi-Myc^+/−^ (**right-panel**) mice. (**C**) Representative pictographs (×100, H&E images) depicting representative histopathological changes in the dorsolateral prostate of *TMPRSS2-ERG. Pten^flox/flox^* (+TAM) and Hi-Myc^+/−^ mice after aspirin and naproxen interventions. Scale bar represents 100 µm. Doses used were aspirin 700 ppm (A700); aspirin 1400 ppm (A1400); naproxen 200 ppm (N200); naproxen 400 ppm (N400). The maximum histological score for the prostate lobe was used to calculate a mean tumor grade for the group. VP, ventral prostate; DLP, dorsolateral prostate; AP, anterior prostate; LGPIN, low-grade prostatic intraepithelial neoplasia; HGPIN, high-grade prostatic intraepithelial neoplasia; MIC, micro-invasive carcinoma; WD, well-differentiated (adenocarcinoma); MD, moderately differentiated (adenocarcinoma); PD, poorly differentiated (adenocarcinoma). Quantified data are represented as Columns (mean for each group): bars represent SEM. *, *p ≤* 0.001; *#, p ≤* 0.01.

With regards to the histopathological evaluation of the harvested prostate, NSAID-fed *TMPRSS2-ERG. Pten^flox/flox^* (+TAM) mice had a significant decrease in the incidence of higher-grade adenocarcinoma lesions compared to no-drug controls (but had a higher incidence of PIN—the nonaggressive pre-neoplastic stage) in DLP (Figure 1A, left panel). Specifically, the incidence of MD adenocarcinoma in the DLP decreased from ~57% (no-drug controls) to ~5% (aspirin 700 ppm) and ~12% (aspirin 1400 ppm), signifying a ~79–91% decrease in MD incidence in the aspirin cohorts (Figure 1C). Markedly, both doses of naproxen seemed to have a slightly stronger preventive effect than aspirin, as indicated by a complete absence of MD tumors in DLP compared to no-drug controls. It may be noted that highly aggressive PD adenocarcinomas were not observed in this mouse model [12]. Importantly, the reduction in the incidence of any type of adenocarcinoma (micro, WD and MD) was significant at both low and high naproxen dose levels but only significant for the low aspirin dose and not the high aspirin dose. High-grade PIN (HGPIN) lesions were concomitantly increased in the NSAID groups; the incidence of HGPIN increased from ~5% (no-drug controls) to ~48% (aspirin 700 ppm), ~32% (aspirin 1400 ppm), ~50% (naproxen 200 ppm), and ~38% (naproxen 400 ppm). This observation indicates the arrest of tumor progression at the lower stages by the NSAIDs evaluated in this study. Notably, WD adenocarcinoma lesions were also significantly decreased by both NSAIDs; specifically, naproxen 200 ppm and naproxen 400 ppm decreased the WD incidence from ~29% to ~5% and ~7%, respectively. However, the DLP effects of these NSAIDs were not dose-related. In the AP lobe, a similar trend was observed; the NSAIDs decreased the incidence of higher-grade adenocarcinoma lesions (MD) from ~26% (no-drug controls) to ~10% (aspirin 700 ppm), ~11% (aspirin 1400 ppm), ~4% (naproxen 200 ppm), and ~7% (naproxen 400 ppm). However, there appeared to be an inverted dose response with the higher dose level of both NSAIDs resulting in a lesser protective effect than the low doses. Since in the VP, not many aggressive pathologies were observed in the non-drug control group; the NSAIDs had no significant impact on their incidence.

**Figure 2:**
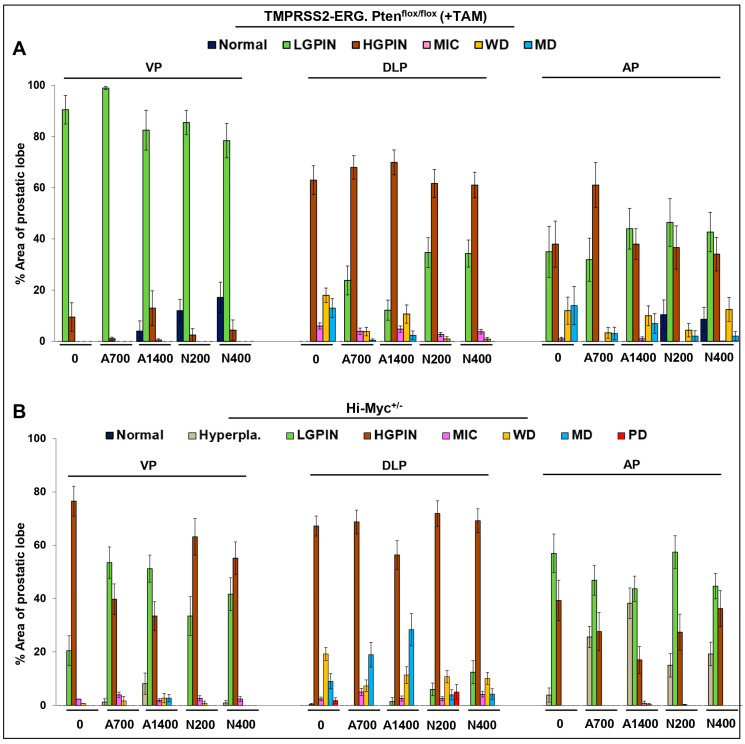
Effects of aspirin and naproxen interventions on % area of lesions in different prostate lobes of *TMPRSS2-ERG* fusion-driven and non-fusion-driven Hi-Myc^+/−^ PCa models. NSAID effects on % area of different prostate lobes displaying normal, pre-/neoplastic, adenocarcinoma lesions in (**A**) *TMPRSS2-ERG. Pten^flox/flox^* (+TAM), and (**B**) Hi-Myc^+/−^ mice. Doses used were aspirin 700 ppm (A700); aspirin 1400 ppm (A1400); naproxen 200 ppm (N200); naproxen 400 ppm (N400). Data are presented as the % area of the prostatic lobe of each group. VP, ventral prostate; DLP, dorsolateral prostate; AP, anterior prostate; LGPIN, low-grade prostatic intraepithelial neoplasia; HGPIN, high-grade prostatic intraepithelial neoplasia; MIC, micro-invasive carcinoma; WD, well-differentiated (adenocarcinoma); MD, moderately differentiated (adenocarcinoma); PD, poorly differentiated (adenocarcinoma). Quantified data are represented as Columns (mean for each group): bars represent SEM. (Statistical data in Supplementary Tables S2 and S3).

In terms of the effect on % area of prostatic lesions (Figure 2A and Supplementary Table S2), there was a decrease in DLP % area covered by MD lesions; while both naproxen dose groups had absolutely no MD lesions, aspirin 700 ppm, and aspirin 1400 ppm interventions decreased the ~13% (no-drug controls) area covered by MD adenocarcinoma lesions to ~ 0.5% and ~2.4%, respectively; a similar trend was observed for WD lesions. In terms of effect on tumor grade (Figure 1B, left panel), in DLP, the tumor grade was decreased from 4.48 ± 0.15 (no-drug controls) to 3.42 ± 0.12 (*p* ≤ 0.001, aspirin 700 ppm), 3.64 ± 0.13 (*p* ≤ 0.001, aspirin 1400 ppm), 3.27 ± 0.06 (*p* ≤ 0.001, naproxen 200 ppm), and 3.29 ± 0.07 (*p* ≤ 0.001, naproxen 400 ppm). Again, no dose-dependent effects were observed in the aspirin and naproxen groups with respect to the efficacy against cancer progression; the high aspirin dose effect was somewhat less than that of the low aspirin dose.

In the Hi-Myc^+/−^ mouse model, all the NSAID diet-fed groups showed little protective effect against prostate tumorigenesis compared to the control diet-fed Hi-Myc^+/−^ group. Even though there was a slight decrease in the incidence of PD adenocarcinoma in DLP (Figure 1A, right panel) (complete absence in aspirin groups and naproxen 400 ppm group), however, this decrease was not considered biologically significant as the average prostate area of the PD lesions in control diet-fed Hi-Myc group was only ~2%; incidentally, naproxen 200 ppm did not affect the incidence of PD significantly (Figure 2B and Supplementary Table S3). Importantly, tumor grade was also only marginally affected in the NSAID-treated groups (in VP, DLP, and AP) (Figure 1B, right panel). Although the naproxen 400 ppm dose effect was slightly more efficacious than the naproxen 200 ppm dose or the aspirin doses, these differences were not statistically significant.

Nonetheless, this differential effect of NSAIDs’ efficacy in fusion-driven versus non-fusion-driven PCa models suggests that NSAIDs have a specific protective effect against *TMPRSS2-ERG* (fusion)-positive PCa, as previously suggested [2].

### 3.2. Differential Effects of Aspirin and Naproxen Intervention on the Expression of Proliferative Markers and ETS Transcription Factor (ERG) in the Prostate of TMPRSS2-ERG Fusion-Driven and Non-Fusion-Driven Hi-Myc^+/-^ PCa Models

**Figure 3:**
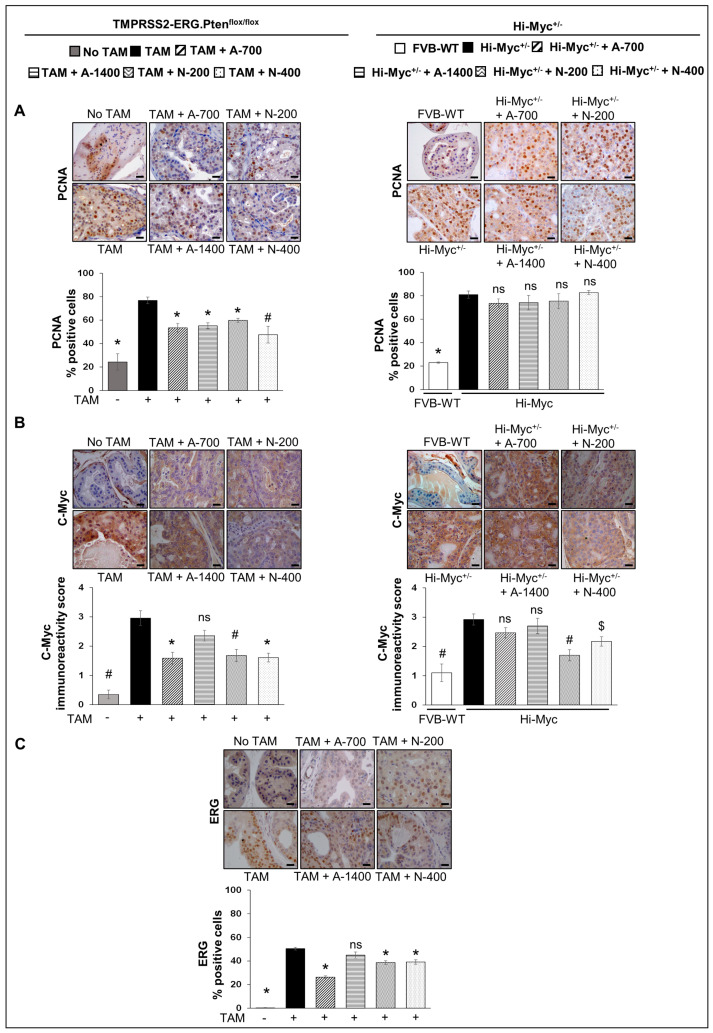
Differential effects of aspirin and naproxen intervention on the expression of proliferative markers and ETS transcription factor (ERG) in the prostate of *TMPRSS2-ERG* fusion-driven and non-fusion-driven Hi-Myc^+/−^ PCa models. NSAID effects on (**A**) PCNA, (**B**) c-Myc in dorsolateral prostate of *TMPRSS2-ERG. Pten^flox/flox^* (+TAM) (**left-panel**), and Hi-Myc^+/−^ (**right-panel**) mice. (**C**) ERG expression in dorsolateral prostate of *TMPRSS2-ERG. Pten^flox/flox^* (+TAM). Representative pictographs (×400 magnification) of DAB-stained prostate tissues showing brown-colored positive staining (PCNA-nuclear, c-Myc-cytoplasmic/nuclear, ERG-nuclear) are shown above each panel. Scale bar represents 20 µm. Doses used were aspirin 700 ppm (A700); aspirin 1400 ppm (A1400); naproxen 200 ppm (N200); naproxen 400 ppm (N400). Age-matched NSAID untreated mice [(No-TAM) and FVB (WT)] represent respective controls for each strain. Quantified data are represented as Columns (mean for each group): [*TMPRSS2-ERG. Pten^flox/flox^* (+TAM) and Hi-Myc^+/−^ (untreated and NSAID-fed) *n* = 10 tissues/group; No-TAM: *n* = 4 tissues; FVB (WT): *n* = 3 tissues; bars represent SEM. *, p ≤ 0.001; *#, p ≤* 0.01; *$, p* ≤ 0.05; ns, *p* > 0.05.

Proliferation cell nuclear antigen (PCNA)**:** IHC analysis for PCNA (proliferative index) revealed that the treatment with NSAIDs (both drugs/doses) significantly decreased the number of PCNA-positive cells in *TMPRSS2-ERG. Pten^flox/flox^* (+TAM) mouse prostate by ~28–30% (*p* ≤ 0.001) in the aspirin groups and ~22–38% (*p* ≤ 0.01–*p* ≤ 0.001) in the naproxen groups, but not in the Hi-Myc^+/−^ mice (Figure 3A). The data strongly suggested that treatment with NSAIDs results in decreased proliferation of PCa tumor cells, specifically in the *TMPRSS2-ERG* fusion-positive mice, and thus, corroborated the histopathological observations, which indicated that the NSAIDs also caused a significant arrest of PCa tumor progression in *TMPRSS2-ERG* fusion-positive mice but not in the Hi-Myc^+/−^ mice.

#### 3.2.1. C-Myc

IHC analysis of the proliferative/oncogenic marker, c-Myc, revealed that the treatment with NSAIDs [naproxen: both 200 and 400 ppm doses (*p* ≤ 0.01 and *p* ≤ 0.001, respectively), aspirin 700 ppm dose (*p* ≤ 0.001)] resulted in significant reduction of c-Myc expression in *TMPRSS2-ERG. Pten^flox/flox^* (+TAM) mouse prostate. In Hi-Myc^+/−^ mice, both doses of naproxen (200 and 400 ppm) substantially reduced (*p* ≤ 0.01–*p* ≤ 0.05) the expression of c-Myc in the prostate tissue, whereas aspirin treatments had no effect (Figure 3B).

#### 3.2.2. ERG

Changes in ERG expression upon treatment with NSAIDs were investigated by IHC analysis, indicating ERG expression in *TMPRSS2-ERG. Pten^flox/flox^* (+TAM) mice PCa tissues was reduced considerably after treatment with NSAIDs [naproxen: both 200 and 400 ppm doses, aspirin 700 ppm dose (*p* ≤ 0.001 for all)]. Treatment with aspirin 1400 ppm dose did not result in any significant decrease in the ERG-positive population (Figure 3C). Since the ERG protein overexpression is likely due to the *TMPRSS2/ERG* fusion [21,22], it is interesting to note that treatment with NSAIDs led to the decreased level of the fusion gene activity, suppressing the overexpression of ERG and possibly the reduced expression of ERG-target genes involved in prostate tumorigenesis. Given that ERG was not overexpressed in the Hi-Myc^+/−^ mice prostate (as reported by us previously) [12], we did not evaluate the impact of NSAIDs on ERG in that cohort.

### 3.3. Differential Effects of Aspirin and Naproxen Intervention on Angiogenesis and Apoptosis Markers in the Prostate of TMPRSS2-ERG Fusion-Driven and Non-Fusion-Driven Hi-Myc^+/−^ PCa Models

#### 3.3.1. CD-31 (PECAM-1)

**Figure 4:**
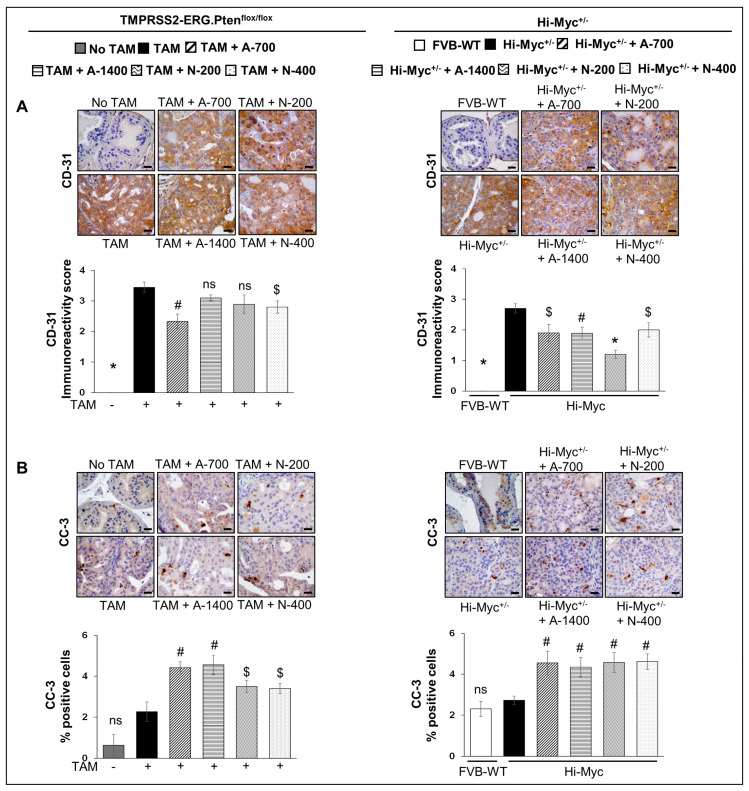
Differential effects of aspirin and naproxen intervention on angiogenesis and apoptosis markers in the prostate of *TMPRSS2-ERG* fusion-driven and non-fusion-driven Hi-Myc PCa models. NSAID effects on (**A**) CD-31 (PECAM-1) angiogenesis marker, and (**B**) cleaved-caspase-3 (apoptosis marker) in dorsolateral prostate of *TMPRSS2-ERG. Pten^flox/flox^* (+TAM) (**left-panel**), and Hi-Myc^+/−^ (**right-panel**) mice. Representative pictographs (×400 magnification) of DAB-stained prostate tissues showing brown-colored positive staining (CD-31: endothelial membrane junction, c-caspase-3: cytoplasmic/nuclear) are shown above each panel. Scale bar represents 20 µm. Doses used were aspirin 700 ppm (A700); aspirin 1400 ppm (A1400); naproxen 200 ppm (N200); naproxen 400 ppm (N400). Age-matched NSAID untreated mice [(No-TAM) and FVB (WT)] represent respective controls for each strain. Quantified data are represented as Columns (mean for each group): [*TMPRSS2-ERG. Pten^flox/flox^* (+TAM) and Hi-Myc^+/−^ (untreated and NSAID-fed) *n* =10 tissues/group; No-TAM: *n* = 4 tissues; FVB (WT): *n* = 3 tissues; bars represent SEM. *, *p* ≤ 0.001; #, *p* ≤ 0.01; $, *p* ≤ 0.05; ns, *p* > 0.05.

IHC analysis for angiogenesis marker CD-31 revealed that both aspirin and naproxen treatments reduced the expression of CD-31 significantly (*p* ≤ 0.05–*p* ≤ 0.001) in the Hi-Myc^+/−^ mouse prostate tissue. However, in the *TMPRSS2-ERG. Pten^flox/flox^* (+TAM) mouse model, aspirin and naproxen treatments were not as effective, and only the lower dose of aspirin (700 ppm, *p* ≤ 0.01) and the higher dose of naproxen (400 ppm, *p* ≤ 0.05) were associated with a significant decrease in CD-31 expression (Figure 4A). Taken together, these results indicate that while NSAIDs exhibit anti-angiogenic benefits, irrespective of *TMPRSS2-ERG* fusion state, the main factor defining their anti-PCa effect does not appear to involve anti-angiogenic activities but rather significant impact on proliferation, especially in the fusion-driven prostate tumorigenesis state (compared to no effect on proliferation in the Hi-Myc^+/−^ mice prostate).

#### 3.3.2. Cleaved Capsase-3

IHC analysis for cleaved caspase-3 was performed to investigate the effect of NSAIDs on apoptosis induction. Both NSAID treatments (both drugs/doses) increased the number of cleaved caspase 3-positive cells in both *TMPRSS2-ERG. Pten^flox/flox^* (+TAM) (~1.5–2 folds, *p* ≤ 0.01–*p* ≤ 0.05) and Hi-Myc^+/−^ mouse prostate (~1.6–1.7 folds, *p* ≤ 0.01) compared to their no-drug controls (Figure 4B). However, the % of apoptotic cells (though significantly increased) was overall very low (~3–5%) in the prostate tissue of these NSAID treated groups in both mouse models.

### 3.4. Differential Effects of Aspirin and Naproxen Intervention on the Cell Type Distribution (Basal, Luminal) in the Prostate of TMPRSS2-ERG Fusion-Driven and Non-Fusion-Driven Hi-Myc^+/−^ PCa Models

#### (CK5/CK8) Basal/Luminal Epithelial Cell Markers

**Figure 5:**
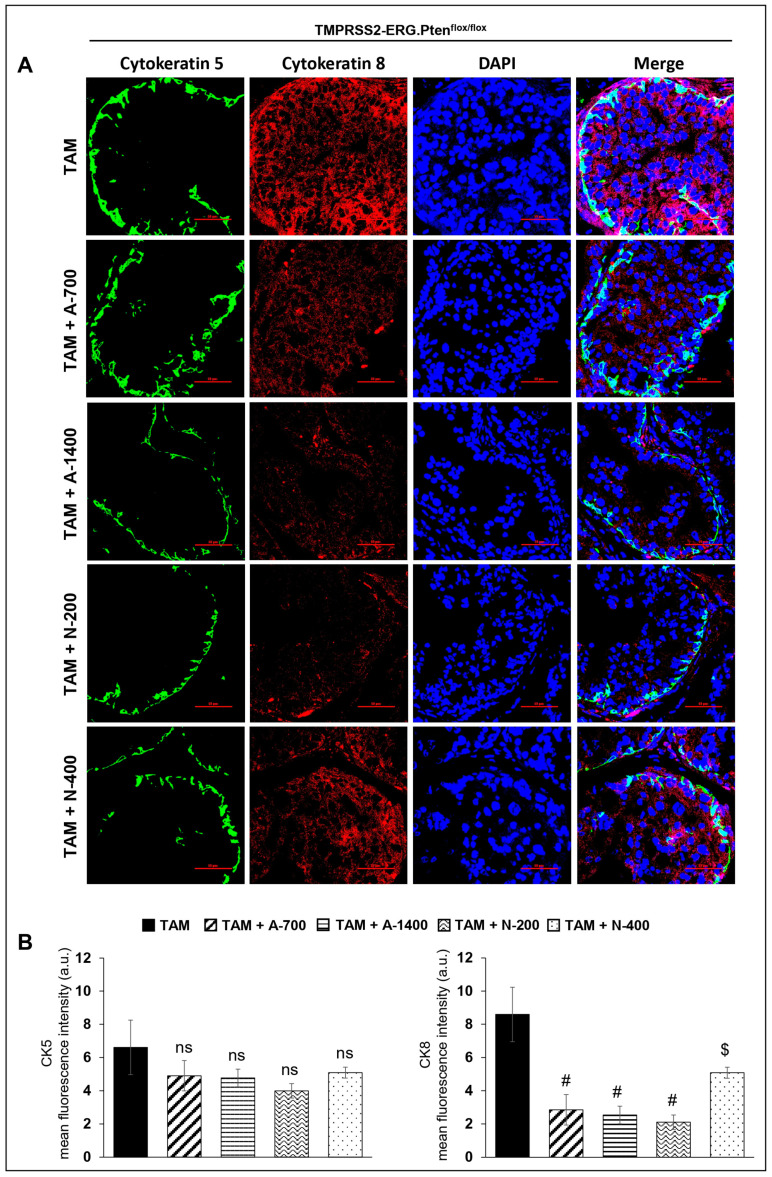

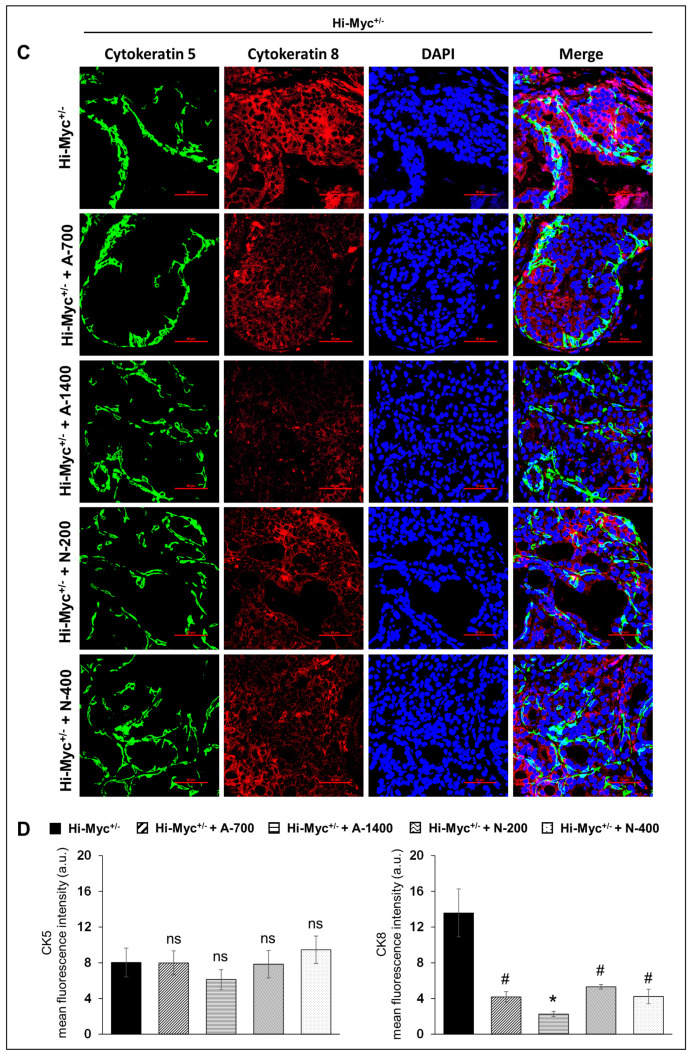
Differential effects of aspirin and naproxen intervention on the cell type distribution (basal, luminal) in the prostate of *TMPRSS2-ERG* fusion-driven and non-fusion-driven Hi-Myc^+/−^ PCa models. NSAID effects on the expression pattern (dual immunofluorescence staining) of basal cell marker CK-5 (green) and luminal cell marker CK-8 (red) in dorsolateral prostate of (**A**) *TMPRSS2-ERG. Pten^flox/flox^* (+TAM), and (**C**) Hi-Myc^+/-^ mice. Nuclei are stained blue with DAPI. Scale bar represents 50 µm. (**B**,**D**) Quantified data for mean fluorescence intensity are represented as Columns [CK-5 (**left panel)** and CK-8 (**right panel**)]: mean for each group from 10 fields (×600) per 2 tissue sections; bars represent SEM. *, p ≤ 0.001; #, *p* ≤ 0.01; $, *p* ≤ 0.05; ns, *p* > 0.05. CK, cytokeratin. Doses used were aspirin 700 ppm (A700); aspirin 1400 ppm (A1400); naproxen 200 ppm (N200); naproxen 400 ppm (N400).

Immunofluorescence (IF) staining to investigate the effect of NSAIDs on epithelial cell type distribution in *TMPRSS2-ERG. Pten^flox/flox^* (+TAM) and Hi-Myc^+/−^ mouse prostates indicated that both NSAIDs caused a significant decrease (*p* ≤ 0.01–*p* ≤ 0.05) in the expression level of the luminal cell marker CK8 but did not change the expression of basal cell marker CK5, suggesting that NSAID treatment significantly changes the mean distribution of the CK5/CK8 population (Figure 5).

### 3.5. Differential Effects of Aspirin and Naproxen Intervention on the Expression of Prostate-Specific Solute Carrier Family Member (Prostein) and Androgen Receptor (AR) in the Prostate of TMPRSS2-ERG Fusion-Driven and Non-Fusion-Driven Hi-Myc^+/−^ PCa Models

#### 3.5.1. Prostein (SLC45A3)

**Figure 6:**
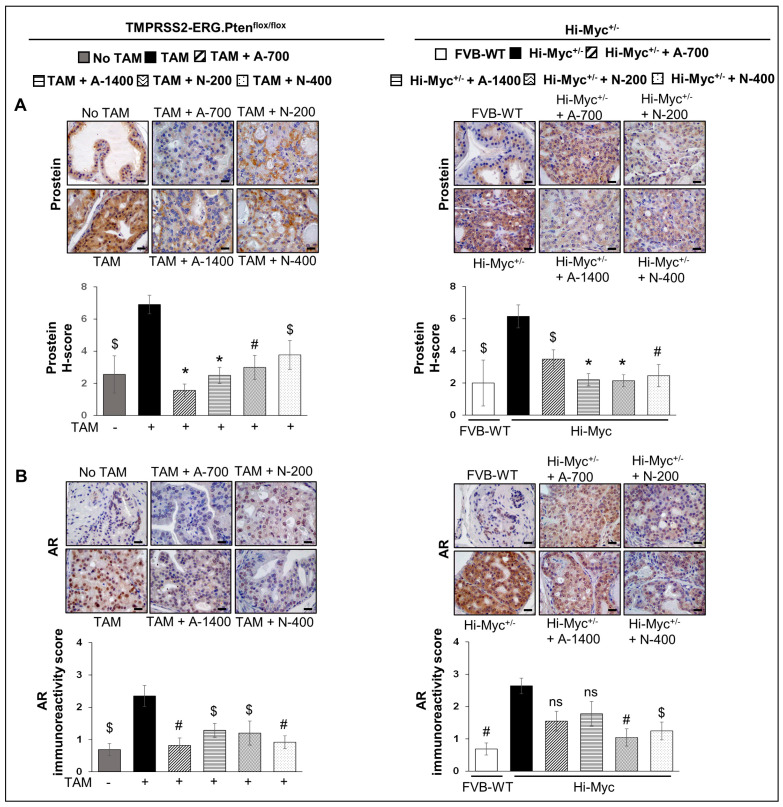
Differential effects of aspirin and naproxen intervention on the expression of prostate-specific solute carrier family member (prostein) and AR in the prostate of *TMPRSS2-ERG* fusion-driven and non-fusion-driven Hi-Myc^+/−^ PCa models. NSAID effects on (**A**) Prostein (SLC45A3), and (**B**) Androgen receptor (AR) expression in dorsolateral prostate of *TMPRSS2-ERG. Pten^flox/flox^* (+TAM) (**left-panel**), and Hi-Myc^+/−^ (**right-panel**) mice. Representative pictographs (×400 magnification) of DAB-stained prostate tissues showing brown-colored positive staining (AR-nuclear intensity and prostein-peri-nuclear presence) are shown above each panel. Scale bar represents 20 µm. Doses used were aspirin 700 ppm (A700); aspirin 1400 ppm (A1400); naproxen 200 ppm (N200); naproxen 400 ppm (N400). Age-matched NSAID untreated mice [(No-TAM) and FVB (WT)] represent respective controls for each strain. Quantified data are represented as Columns (mean for each group): [*TMPRSS2-ERG. Pten^flox/flox^* (+TAM) and Hi-Myc^+/−^ (untreated and NSAID-fed) *n* = 10 tissues/group; No-TAM: *n* = 4 tissues; FVB (WT): *n* = 3 tissues; bars represent SEM. *, p ≤ 0.001; #, *p* ≤ 0.01; $, *p* ≤ 0.05; ns, *p* > 0.05.

IHC analysis for prostein (SLC45A3) revealed that NSAID treatments (both drugs/doses) significantly reduced the H-score of prostein expression in prostate tissues in both *TMPRSS2-ERG. Pten^flox/flox^* (+TAM) and Hi-Myc^+/−^ mouse models (*p* ≤ 0.05–*p* ≤ 0.001 for both) (Figure 6A). Prostein is a prostate-specific protein expressed in normal and malignant prostate tissues [23,24]. The loss of prostein expression in the prostate is associated with SLC45A3-ERG gene fusions and unfavorable clinical outcomes [23,24]; however, in our previously published study [12], we reported overexpression of prostein in both *TMPRSS2-ERG. Pten^flox/flox^* (+TAM) and Hi-Myc^+/−^ mouse models (regardless of the fusion-driven or non-fusion-driven PCa). The clinical significance of our current observation could not be ascertained nor correlated with the differential effects of NSAIDs against fusion-driven vs. non fusion-driven prostate tumorigenesis in our study.

#### 3.5.2. AR

IHC for AR expression revealed that NSAIDs (both drugs/doses) were effective in significantly reducing (*p* ≤ 0.01–*p* ≤ 0.05) the expression levels of AR in *TMPRSS2-ERG. Pten^flox/flox^* (+TAM) mouse prostate by ~46–66% (in aspirin groups) and ~49–61% (in naproxen groups). In Hi-Myc^+/−^ mouse prostate, both doses of naproxen (200 and 400 ppm) significantly reduced the AR expression by ~61% (*p* ≤ 0.01) and ~53% (*p* ≤ 0.05), respectively. In contrast, aspirin treatment (both doses) was not associated with a significant decrease in AR expression (Figure 6B).

### 3.6. Differential Effects of Aspirin and Naproxen Interventions on the Expression of Inflammation-Related Markers in the Prostate of TMPRSS2-ERG Fusion-Driven and Non-Fusion-Driven Hi-Myc^+/−^ PCa Models

#### 3.6.1. NFκb (Total p65)

**Figure 7:**
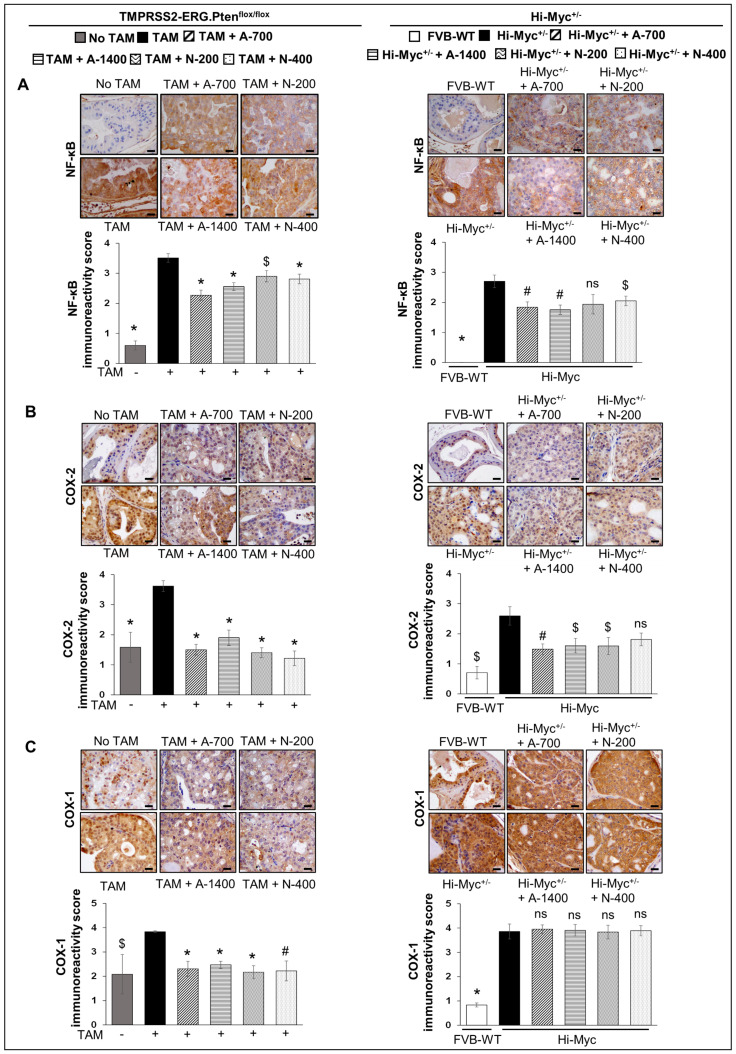
Differential effects of aspirin and naproxen intervention on the expression of inflammation-related markers in the prostate of *TMPRSS2-ERG* fusion-driven and non-fusion-driven Hi-Myc PCa models. NSAID effects on (**A**) NFκB (total p65), (**B**) COX-2, and (**C**) COX-1 expression in dorsolateral prostate of *TMPRSS2-ERG. Pten^flox/flox^* (+TAM) (**left-panel**), and Hi-Myc^+/−^ (**right-panel**) mice. Representative pictographs (×400 magnification) of DAB-stained prostate tissues showing brown-colored positive staining (NFκB—cytoplasmic/nuclear intensity, COX-2, and COX-1: cytoplasmic intensity) are shown above each panel. Scale bar represents 20 µm. Doses used were aspirin 700 ppm (A700); aspirin 1400 ppm (A1400); naproxen 200 ppm (N200); naproxen 400 ppm (N400). Age matched-NSAID untreated mice [(No-TAM) and FVB (WT)] represent respective controls for each strain. Quantified data are represented as Columns (mean for each group): [*TMPRSS2-ERG. Pten^flox/flox^* (+TAM) and Hi-Myc^+/−^ (untreated and NSAID-fed) *n* = 10 tissues/group; No-TAM: *n* = 4 tissues; FVB (WT): *n* = 3 tissues; bars represent SEM. *, p ≤ 0.001; #, *p* ≤ 0.01; $, *p* ≤ 0.05; ns, *p* > 0.05.

IHC analysis indicated that NSAID treatments (both drugs/doses) significantly decreased the expression of NFκb (total p65) in *TMPRSS2-ERG. Pten^flox/flox^* (+TAM) mouse prostate by ~27–35% (*p* ≤ 0.001) in aspirin groups and ~17–20% (*p* ≤ 0.05–*p* ≤ 0.001) in naproxen groups. In Hi-Myc^+/−^ mice, aspirin (700 and 1400 ppm doses) and naproxen (400 ppm dose) decreased the expression of NFκb (total p65) by ~32–35% (*p* ≤ 0.01 for both) and ~24% (*p* ≤ 0.05), respectively (Figure 7A).

#### 3.6.2. COX-1 and COX-2

NSAID treatments (both drugs/doses) significantly decreased (*p* ≤ 0.001) the expression of COX-2 in *TMPRSS2-ERG. Pten^flox/flox^* (+TAM) mouse prostate. Notably, prostate COX-2 expression in *TMPRSS2-ERG. Pten^flox/flox^* (+TAM) mice was reduced by ~47–58% in aspirin groups and ~61–66% in naproxen groups. In Hi-Myc^+/−^ mice, aspirin [700 ppm (*p* ≤ 0.01) and 1400 ppm dose (*p* ≤ 0.05)] and naproxen (200 ppm dose, *p* ≤ 0.05) were associated with the decreased expression (~36–41%, and ~28%, respectively) of COX-2. IHC analysis of COX-1 expression indicated that NSAIDs (both drugs/doses) significantly decreased the expression (*p* ≤ 0.01- *p* ≤ 0.001) only in *TMPRSS2-ERG. Pten^flox/flox^* (+TAM) prostate but not in the Hi-Myc^+/−^ mice (Figure 7B,C).

### 3.7. Differential Effects of Aspirin and Naproxen Intervention on the Expression of Epithelial to Mesenchymal Transition Markers in the Prostate of TMPRSS2-ERG Fusion-Driven and Non-Fusion-Driven Hi-Myc^+/−^ PCa Models

#### E-Cadherin and Vimentin

**Figure 8:**
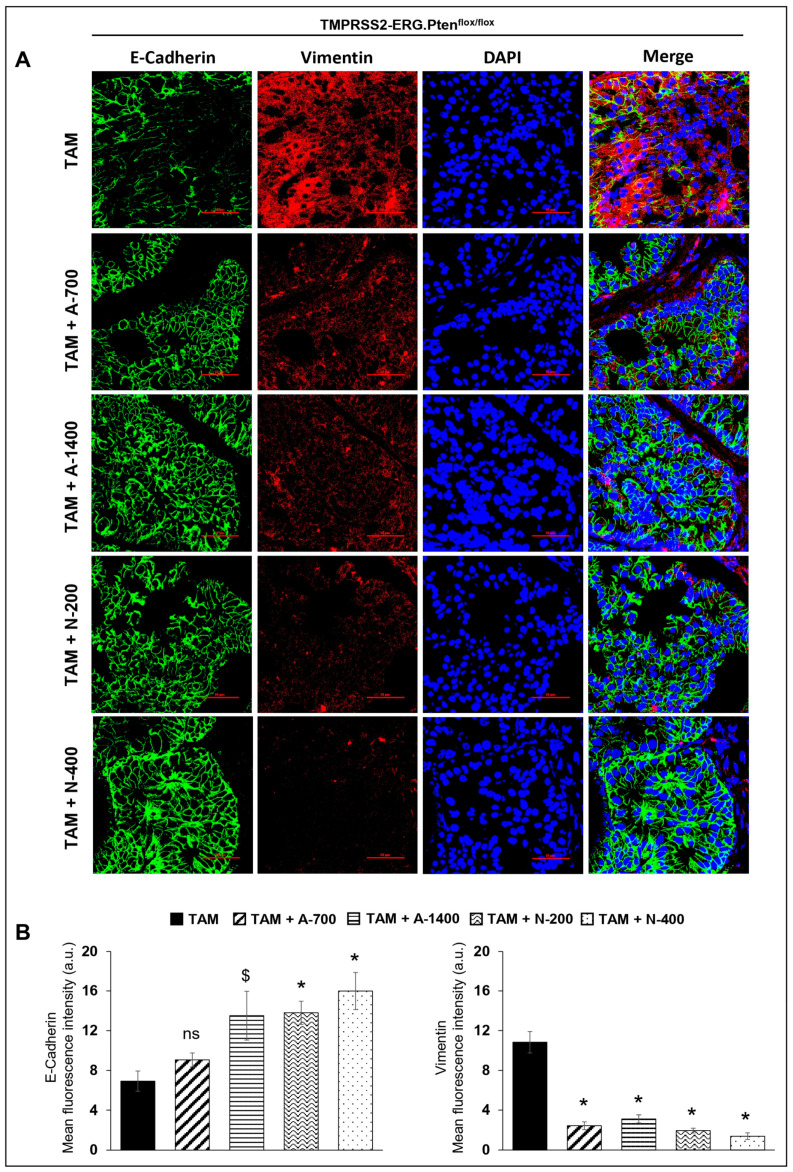

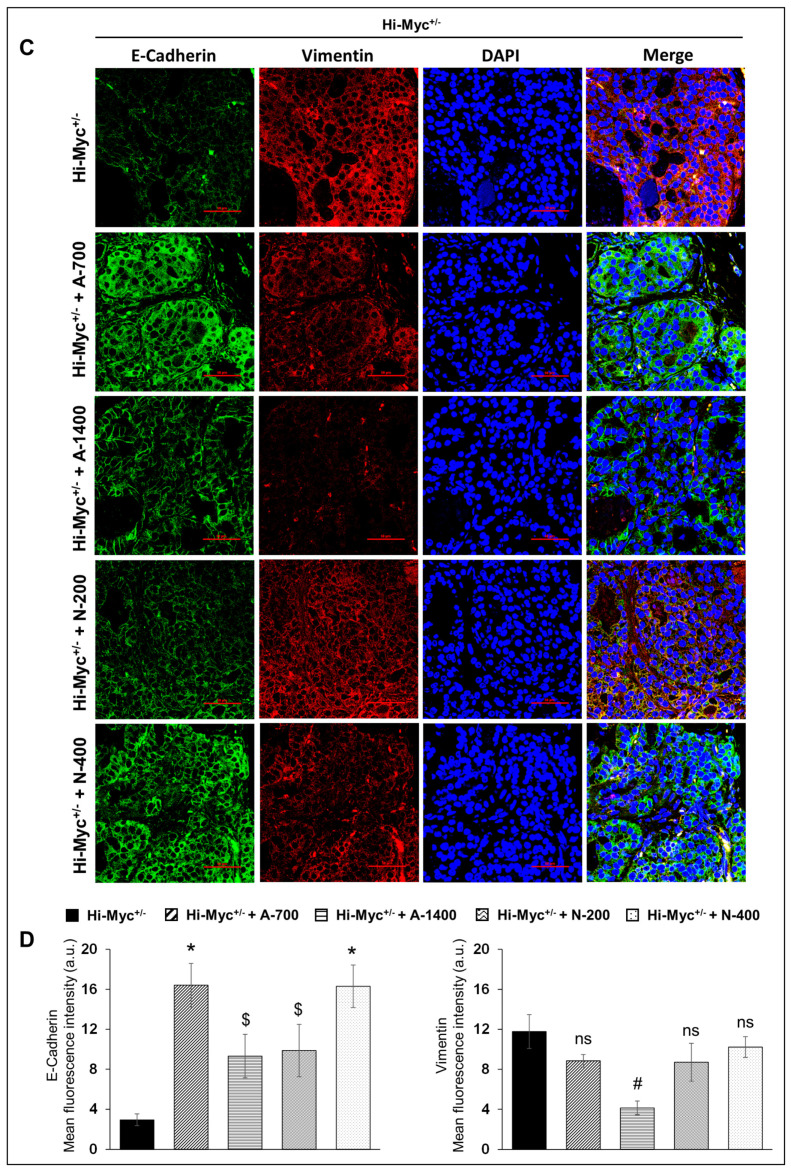
Differential effects of aspirin and naproxen intervention on the expression of epithelial to mesenchymal transition markers in the prostate of *TMPRSS2-ERG* fusion-driven and non-fusion-driven Hi-Myc^+/−^ PCa models. NSAID effects on the expression pattern (dual immunofluorescence staining) of E-cadherin (green) and vimentin (red) in dorsolateral prostate of (**A**) *TMPRSS2-ERG. Pten^flox/flox^* (+TAM), and (**C**) Hi-Myc^+/−^ mice. Nuclei are stained blue with DAPI. Scale bar represents 50 µm. (**B**,**D**) Quantified data for mean fluorescence intensity are represented as Columns [E-cadherin (**left panel**) and vimentin (**right panel**)]: mean for each group from 10 fields (×600) per 2 tissue sections; bars represent SEM. *, p ≤ 0.001; #, *p* ≤ 0.01; $, *p* ≤ 0.05; ns, *p* > 0.05. Doses used were aspirin 700 ppm (A700); aspirin 1400 ppm (A1400); naproxen 200 ppm (N200); naproxen 400 ppm (N400).

IF staining was performed to investigate the effect of NSAIDs on epithelial-mesenchymal markers in *TMPRSS2-ERG. Pten^flox/flox^* (+TAM) and Hi-Myc^+/−^ mouse prostate. Results indicated that the treatment with NSAIDs (both drugs/doses) significantly decreased (*p* ≤ 0.001) the expression of mesenchymal marker vimentin in *TMPRSS2-ERG. Pten^flox/flox^* (+TAM) mouse prostate. Concomitantly, there was an increase in the expression of epithelial marker E-cadherin in *TMPRSS2-ERG. Pten^flox/flox^* (+TAM) by aspirin (1400 ppm dose, *p* ≤ 0.05) and naproxen [200 and 400 pm doses, (*p* ≤ 0.001 for both)]. Treatment with aspirin 700 ppm dose also increased the expression of E-cadherin, although it did not reach statistical significance. In the Hi-Myc^+/−^ mouse prostate, vimentin expression was only decreased by aspirin 1400 ppm dose (*p* ≤ 0.01), while all NSAIDs (both drugs/doses) significantly increased (*p* ≤ 0.05–*p* ≤ 0.001) E-cadherin expression (Figure 8). Taken together, the results suggested that NSAID treatments decreased epithelial to mesenchymal transition of prostate tumor cells irrespective of the *TMPRSS2-ERG* fusion state in PCa.

## 4. Discussion

The American Cancer Society statistical estimates indicate that in 2023, there will be 288,300 new PCa cases and ~34,700 deaths associated with PCa [25]. While a few natural and synthetic drug strategies showed preventive and therapeutic benefits against PCa in preclinical models, most of these agents failed to replicate the protective benefits in clinical studies [26,27,28,29]. One major limitation of clinical prevention studies could be that it takes a considerably long time (decades) for precursor PCa lesions to advance to clinically overt disease stages. As such, it is critical to identify a clinical cohort in which PCa-specific regimens can be evaluated for PCa preventive efficacy long before there are any signs of the disease. Several population-based studies have previously shown a potential relationship between long-term usage/intake of specific drugs and PCa incidence and severity. Interestingly, some of these PCa studies, while focusing on the same agent, have often shown conflicting results [3,27,28,30,31,32,33,34,35]. This suggests the impact of different study populations and thus highlights the need for more specific target cohort selection strategies that have the ability to stratify more homogeneous subsets of individuals at risk for PCa, as heterogeneity of this disease (in clinical/molecular features) can significantly impact the study outcomes [36].

One notable scientific breakthrough in recent times that has the potential to overcome the observed disparity in data outcomes due to study cohort heterogeneity is the identification of PCa fusion genes involving the promoter of transmembrane protease serine 2 (TMPRSS2-prostate-specific and androgen-responsive) fused with the coding sequence of Ets gene family members as oncogenic drivers [36,37,38]. The most common of these fusions is with ERG, an Ets family member, resulting in *TMPRSS2-ERG* fusions, identified in approximately 50% of PCa cases [36,37,38]. Under the control of androgen-sensitive-promoter elements of *TMPRSS2*, the resultant gene fusion leads to the expression of Ets transcription factors [36]. Thus, these gene fusions, specifically *TMPRSS2-ERG* fusions, could be used as targets for effective prevention/intervention strategies and stratification of homogenous PCa patient populations in drug efficacy trials [36,37]. This is indeed an important discovery, as it could help us revisit the efficacy outcomes of potential anti-PCa drugs from a new perspective; specifically if differential outcomes have been reported in clinical trials carried out at different centers, such as those reported for NSAIDs use in PCa [35,39].

For the past several years, pre-clinical studies have strongly supported the protective benefits of NSAIDs for PCa prevention [5]. However, results from epidemiological and clinical studies have been inconclusive. While some studies reported an inverse relationship between NSAID use and PCa risk, other population-based studies have shown either no benefits or increased PCa risk [3,30,31,32,33,34,35]. For example, the Finnish Prostate Cancer Screening trial, which examined NSAID use and PCa risk in 78,615 men, reported increased PCa risk in current NSAID users, while previous use of NSAIDs, including aspirin use, was not associated with PCa [35]. Similarly, in the VITamins And Lifestyle cohort study [30], no significant association between low-dose/regular dose aspirin or other non-aspirin NSAIDs use in the previous decade and PCa risk was observed, although there was a trend towards an inverse association between regular dose aspirin intake and risk of high-grade PCa [30]. On the other hand, the Cancer Prevention Study II Nutrition Cohort reported that daily aspirin use for at least 5 years was associated with a 15% risk reduction of PCa [31]. This is in agreement with another study which reported a significant correlation between daily aspirin intake (past 1 year) and lower risk of highly aggressive PCa tumors (Gleason score ≥7 or Stage III or IV PCa) and between different NSAID use and lower risk of PCa tumors with a Gleason score of 7 or higher [3]. Interestingly, a combination strategy of weekly calcitriol (45 μg per week) and daily naproxen (375 mg, twice daily) in a phase II trial of PCa patients, who had a biochemical recurrence based on increasing prostate-specific antigen (PSA) levels, showed an increase in PSA doubling time in 75% of the patients [40]. A population-based case–control study EPICAP conducted in France that enrolled newly diagnosed PCa patients (<75 years of age) and age-matched male controls demonstrated a significant association between NSAID use and reduced PCa risk (~23% reduction in PCa risk) [3]. Specifically, non-aspirin NSAID use showed protective benefits against aggressive PCa, including PCa associated with prior history of prostatitis [3]. Several meta-analysis studies [32], which have been conducted to investigate these conflicting results, have affirmed the positive benefits of NSAID use against PCa (i.e., NSAID use is inversely related to PCa incidence and PCa-specific mortality) and have attributed the negative results to a lack of data on drug dosage/duration of use.

Interestingly and highly relevant to the present study are the outcomes from a population-based case–control study which reported that aspirin users showed a ~37% reduction in the risk of developing *TMPRSS2-ERG-*positive PCa, and that the risk reduction was stronger with a longer duration of aspirin use [2]. Notably, the study reported no association of aspirin use with *TMPRSS2-ERG-*negative PCa [2]. This is an important finding, as it brings into focus the importance of patient stratification based on *TMPRSS2-ERG* fusion positivity, which was not known earlier and thus was not a selection criterion in patient accrual in the previous trials or case studies looking into NSAID efficacy against PCa. In light of this background, we recently characterized and compared the stage-specific progression of PCa under both *TMPRSS2-ERG* fusion-driven and non-fusion-driven states [12] so that a relevant pre-clinical model could be utilized to address some of the unanswered questions related to the efficacy of NSAIDs against PCa. In that study, we reported that the time-based growth and progression events occur at different rates in the fusion-driven and non-fusion-driven PCa models [12]. Another important outcome from the previous characterization study was a significant increase in the infiltration of immune cells in the *TMPRSS2-ERG* fusion-positive tumors, including a stage-specific increase, compared to fusion-negative tumors in preclinical PCa mouse models [12]. This observation is supported by the fact that previous preclinical in vitro studies have reported the induction of *TMPRSS2-ERG* gene rearrangements in *TMPRSS2-ERG* fusion-negative PCa cells by stress, such as exposure to inflammatory cytokines, oxidative stress, and ionizing radiation [41,42,43]. Given that fusion-driven PCa could be associated with significant inflammatory triggers arising from the immune-rich tumor niche, there lies the possibility that NSAID drugs, such as aspirin and other non-aspirin NSAIDs, due to their ani-inflammatory effect, could be more effective in *TMPRSS2-ERG* fusion-positive PCa. This hypothesis also strongly supports the previous clinical observations that NSAIDs have protective benefits against PCa associated with prostatitis.

Notably, in the present study, treatment with both NSAIDs, aspirin and naproxen, showed strong efficacy in reducing prostate tumorigenesis in the *TMPRSS2-ERG*-driven PCa model (although there was no dose-dependent effect), but not in the non-*TMPRSS2-ERG*-driven (Hi-Myc^+/−^ mouse) PCa model. The lack of dose-dependent effect indicates that an optimum effect against fusion-driven PCa is achievable with lower doses of both NSAIDs and that increasing the doses may not necessarily increase efficacy. From the molecular analysis (IHC-based evaluation) of the prostate tissue samples, differential molecular effects of NSAIDs treatment in both mouse models were observed; however, the biological basis of the strong efficacy of both aspirin and naproxen treatments in reducing prostate tumorigenesis in only the *TMPSS2-ERG* fusion-driven PCa model could not be ascertained. Given that the promoter elements of *TMPRSS2* are androgen-sensitive and that as a result of *TMPRSS2-ERG* fusion, the androgen-bound androgen receptor binds to TMPRSS2 regions, and the downstream cascade of events is initiated for ERG overexpression [36]; thus, there is a considerable likelihood that the NSAID-mediated decrease in AR expression could also be an important factor impacting the inhibitory effect of NSAIDs on fusion-driven PCa. Both NSAIDs exhibited anti-angiogenic benefits irrespective of the *TMPRSS2-ERG*-fusion state. Still, the main factor defining their anti-PCa effects, relative to *TMPRSS2-ERG*-fusion positivity, appears to be the ability of the NSAIDs to significantly affect the proliferative growth phase of the fusion-positive tumors. There is also a possibility that the lack of efficacy in the Hi-Myc model may be due to the inability of NSAIDs to interfere with the molecular mechanisms underlying PCa carcinogenesis in that model. However, the possibility that the very strong tumorigenic stimulus of the overexpression of Myc in Hi-Myc^+/−^ mouse could result in insensitivity to NSAID-inhibition of PCa formation is negated by the fact that there is also increased expression of c-Myc in *TMPRSS2-ERG*-driven PCa model [12]. Results also indicated that even though NSAID treated tumors showed relatively higher apoptotic cells compared to untreated tumors, the apoptotic cells were overall very miniscule in number; thus, the anti-PCa effect exhibited against *TMPRSS2-ERG* fusion-positive tumors by the NSAIDs did not involve apoptosis.

Importantly, compared to aspirin, naproxen showed relatively more protective benefits against *TMPSS2-ERG* fusion-driven prostate tumorigenesis. This could be attributed, at least in part, to its more significant inhibitory effect on the inflammatory trigger molecule (COX-2), as COX-2 has been reported to be overexpressed in PCa, and its higher/aberrant expression has been implicated in PCa growth and progression as well as poor prognosis [6,7]. Among the inflammatory markers studied here, a strong differential inhibition was observed for COX-1. In the Hi-Myc model, no effect was observed for either NSAIDs. This contrasts with the strong inhibition of COX-2 seen for both NSAIDs in both models. Further studies on this pathway may shed light on the molecular mechanism for this effect. Additional studies are warranted to critically assess the effects of the NSAIDs on inflammatory signaling pathways and inflammatory/immune cell type infiltration-subtype in the prostate tissue and to elucidate the mechanistic reason behind the preventive efficacy of NSAIDs and seemingly superior efficacy of non-aspirin NSAID naproxen compared to aspirin in the *TMPSS2-ERG*-driven PCa model, but not in the non-*TMPRSS2-ERG*-driven PCa model.

## 5. Conclusions

Overall, the study outcomes from the present study are the first of its kind to indicate the benefits of NSAIDs, specifically against *TMPSS2-ERG* fusion-driven prostate tumorigenesis, which could help in patient stratification in future PCa clinical trials. Although there were some differential molecular effects of NSAID treatments in both mouse models, these molecular changes could not collectively establish the biological basis of the strong efficacy of both aspirin and naproxen NSAID treatments in reducing PCa tumorigenesis in only the *TMPSS2-ERG* fusion-driven prostate cancer model and not in the non-fusion-driven PCa model.

## Data Availability

The data presented in this study are available in Figures within the Manuscript or as supplementary material. The data presented in this study are also available on request from the corresponding author.

## Supplementary Materials

The following supporting information can be downloaded at: https://www.mdpi.com/article/10.3390/cancers15205054/s1, Figure S1. Effect of aspirin and naproxen intervention on the lower urogenital tract (LUT) weight (normalized: g/100 g of body weight) of *TMPRSS2-ERG*; Figure S2. Representative pictographs (×100, H&E images) depicting representative histopathological changes in the anterior and ventral prostate of TMPRSS2-ERG; Figures S3–S6. NSAID-feeding effects on PCNA, cleaved caspase-3, c-Myc, Androgen receptor (AR), ERG, prostein (SLC45A3), CD-31 (PECAM-1), NFκB (total p65), COX-2, and COX-1 expression in the dorsolateral prostate of *TMPRSS2-ERG*; Table S1. Experimental groups to determine the preventive efficacy of aspirin and naproxen in the *TMPRSS2-ERG* fusion-driven and non-fusion-driven models of prostate cancer (PCa); Table S2. *TMPRSS2.ERG*. *Pten^flox/flox^* (+TAM); Table S3. Hi-Myc^+/−^ mice.

## Abbreviations

PCa	prostate cancer
*TMPRSS2*	transmembrane protease serine 2
Ets	erythroblastosis virus E26
ERG	Ets related gene
TAM	tamoxifen
LUT	lower urogenital tract
PIN	prostatic intraepithelial neoplasia
LGPIN	low-grade PIN
HGPIN	high-grade PIN
WD	well differentiated
MD	moderately differentiated
PD	poorly differentiated
PCNA	proliferation cell nuclear antigen
PECAM-1/CD-31	platelet endothelial cell adhesion molecule-1
DAB	3,3′-diaminobenzidine
IHC	immunohistochemistry

## References

[1] Salinas C.A., Kwon E.M., FitzGerald L.M., Feng Z., Nelson P.S., Ostrander E.A., Peters U., Stanford J.L. (2010). Use of aspirin and other nonsteroidal anti-inflammatory medications in relation to prostate cancer risk. Am. J. Epidemiol..

[2] Wright J.L., Chery L., Holt S., Lin D.W., Luedeke M., Rinckleb A.E., Maier C., Stanford J.L. (2016). Aspirin and NSAID use in association with molecular subtypes of prostate cancer defined by TMPRSS2:ERG fusion status. Prostate Cancer Prostatic. Dis..

[3] Doat S., Cenee S., Tretarre B., Rebillard X., Lamy P.J., Bringer J.P., Iborra F., Murez T., Sanchez M., Menegaux F. (2017). Nonsteroidal anti-inflammatory drugs (NSAIDs) and prostate cancer risk: Results from the EPICAP study. Cancer Med..

[4] Alfonso L., Ai G., Spitale R.C., Bhat G.J. (2014). Molecular targets of aspirin and cancer prevention. Br. J. Cancer.

[5] Ishiguro H., Kawahara T. (2014). Nonsteroidal anti-inflammatory drugs and prostatic diseases. Biomed. Res. Int..

[6] Shao N., Feng N., Wang Y., Mi Y., Li T., Hua L. (2012). Systematic review and meta-analysis of COX-2 expression and polymorphisms in prostate cancer. Mol. Biol. Rep..

[7] Kirschenbaum A., Liu X., Yao S., Levine A.C. (2001). The role of cyclooxygenase-2 in prostate cancer. Urology.

[8] Taverna G., Pedretti E., Di Caro G., Borroni E.M., Marchesi F., Grizzi F. (2015). Inflammation and prostate cancer: Friends or foe?. Inflamm. Res..

[9] McGettigan P., Henry D. (2011). Cardiovascular risk with non-steroidal anti-inflammatory drugs: Systematic review of population-based controlled observational studies. PLoS Med..

[10] King J.C., Xu J., Wongvipat J., Hieronymus H., Carver B.S., Leung D.H., Taylor B.S., Sander C., Cardiff R.D., Couto S.S. (2009). Cooperativity of TMPRSS2-ERG with PI3-kinase pathway activation in prostate oncogenesis. Nat. Genet..

[11] Gao D., Zhan Y., Di W., Moore A.R., Sher J.J., Guan Y., Wang S., Zhang Z., Murphy D.A., Sawyers C.L. (2016). A Tmprss2-CreERT2 Knock-In Mouse Model for Cancer Genetic Studies on Prostate and Colon. PLoS ONE.

[12] Raina K., Kant R., Prasad R.R., Kandhari K., Tomar M., Mishra N., Kumar R., Fox J.T., Sei S., Shoemaker R.H. (2022). Characterization of stage-specific tumor progression in TMPRSS2-ERG (fusion)-driven and non-fusion-driven prostate cancer in GEM models. Mol. Carcinog..

[13] Akinyeke T., Matsumura S., Wang X., Wu Y., Schalfer E.D., Saxena A., Yan W., Logan S.K., Li X. (2013). Metformin targets c-MYC oncogene to prevent prostate cancer. Carcinogenesis.

[14] Ellwood-Yen K., Graeber T.G., Wongvipat J., Iruela-Arispe M.L., Zhang J., Matusik R., Thomas G.V., Sawyers C.L. (2003). Myc-driven murine prostate cancer shares molecular features with human prostate tumors. Cancer Cell.

[15] Saha A., Blando J., Tremmel L., DiGiovanni J. (2015). Effect of Metformin, Rapamycin, and Their Combination on Growth and Progression of Prostate Tumors in HiMyc Mice. Cancer Prev. Res..

[16] Mohammed A., Janakiram N.B., Madka V., Zhang Y., Singh A., Biddick L., Li Q., Lightfoot S., Steele V.E., Lubet R.A. (2019). Intermittent Dosing Regimens of Aspirin and Naproxen Inhibit Azoxymethane-Induced Colon Adenoma Progression to Adenocarcinoma and Invasive Carcinoma. Cancer Prev. Res..

[17] Raina K., Blouin M.J., Singh R.P., Majeed N., Deep G., Varghese L., Glode L.M., Greenberg N.M., Hwang D., Cohen P. (2007). Dietary feeding of silibinin inhibits prostate tumor growth and progression in transgenic adenocarcinoma of the mouse prostate model. Cancer Res..

[18] Raina K., Rajamanickam S., Singh R.P., Deep G., Chittezhath M., Agarwal R. (2008). Stage-specific inhibitory effects and associated mechanisms of silibinin on tumor progression and metastasis in transgenic adenocarcinoma of the mouse prostate model. Cancer Res..

[19] Shappell S.B., Thomas G.V., Roberts R.L., Herbert R., Ittmann M.M., Rubin M.A., Humphrey P.A., Sundberg J.P., Rozengurt N., Barrios R. (2004). Prostate pathology of genetically engineered mice: Definitions and classification. The consensus report from the Bar Harbor meeting of the Mouse Models of Human Cancer Consortium Prostate Pathology Committee. Cancer Res..

[20] Raina K., Ravichandran K., Rajamanickam S., Huber K.M., Serkova N.J., Agarwal R. (2013). Inositol hexaphosphate inhibits tumor growth, vascularity, and metabolism in TRAMP mice: A multiparametric magnetic resonance study. Cancer Prev. Res..

[21] Chen Y., Chi P., Rockowitz S., Iaquinta P.J., Shamu T., Shukla S., Gao D., Sirota I., Carver B.S., Wongvipat J. (2013). ETS factors reprogram the androgen receptor cistrome and prime prostate tumorigenesis in response to PTEN loss. Nat. Med..

[22] Sung J.Y., Jeon H.G., Jeong B.C., Seo S.I., Jeon S.S., Lee H.M., Choi H.Y., Kang S.Y., Choi Y.L., Kwon G.Y. (2016). Correlation of ERG immunohistochemistry with molecular detection of TMPRSS2-ERG gene fusion. J. Clin. Pathol..

[23] Hernandez-Llodra S., Juanpere N., de Muga S., Lorenzo M., Gil J., Font-Tello A., Agell L., Albero-Gonzalez R., Segales L., Merino J. (2017). ERG overexpression plus SLC45A3 (prostein) and PTEN expression loss: Strong association of the triple hit phenotype with an aggressive pathway of prostate cancer progression. Oncotarget.

[24] Perner S., Rupp N.J., Braun M., Rubin M.A., Moch H., Dietel M., Wernert N., Jung K., Stephan C., Kristiansen G. (2013). Loss of SLC45A3 protein (prostein) expression in prostate cancer is associated with SLC45A3-ERG gene rearrangement and an unfavorable clinical course. Int. J. Cancer.

[25] Siegel R.L., Miller K.D., Wagle N.S., Jemal A. (2023). Cancer statistics, 2023. CA Cancer J. Clin..

[26] Matsushita M., Fujita K., Nonomura N. (2020). Influence of Diet and Nutrition on Prostate Cancer. Int. J. Mol. Sci..

[27] Reed D., Raina K., Agarwal R. (2018). Nutraceuticals in prostate cancer therapeutic strategies and their neo-adjuvant use in diverse populations. NPJ Precis. Oncol..

[28] Hamilton Z., Parsons J.K. (2016). Prostate Cancer Prevention: Concepts and Clinical Trials. Curr. Urol. Rep..

[29] Singh R.P., Agarwal R. (2006). Mechanisms of action of novel agents for prostate cancer chemoprevention. Endocr. Relat. Cancer.

[30] Brasky T.M., Velicer C.M., Kristal A.R., Peters U., Potter J.D., White E. (2010). Nonsteroidal anti-inflammatory drugs and prostate cancer risk in the VITamins And Lifestyle (VITAL) cohort. Cancer Epidemiol. Biomark. Prev..

[31] Jacobs E.J., Thun M.J., Bain E.B., Rodriguez C., Henley S.J., Calle E.E. (2007). A large cohort study of long-term daily use of adult-strength aspirin and cancer incidence. J. Natl. Cancer Inst..

[32] Liu Y., Chen J.Q., Xie L., Wang J., Li T., He Y., Gao Y., Qin X., Li S. (2014). Effect of aspirin and other non-steroidal anti-inflammatory drugs on prostate cancer incidence and mortality: A systematic review and meta-analysis. BMC Med..

[33] Mahmud S.M., Franco E.L., Turner D., Platt R.W., Beck P., Skarsgard D., Tonita J., Sharpe C., Aprikian A.G. (2011). Use of non-steroidal anti-inflammatory drugs and prostate cancer risk: A population-based nested case-control study. PLoS ONE.

[34] Skriver C., Dehlendorff C., Borre M., Brasso K., Sorensen H.T., Hallas J., Larsen S.B., Tjonneland A., Friis S. (2016). Low-dose aspirin or other nonsteroidal anti-inflammatory drug use and prostate cancer risk: A nationwide study. Cancer Causes Control.

[35] Veitonmaki T., Murtola T.J., Maattanen L., Taari K., Stenman U.H., Tammela T.L., Auvinen A. (2014). Prostate cancer risk and nonsteroidal antiinflammatory drug use in the Finnish prostate cancer screening trial. Br. J. Cancer.

[36] St John J., Powell K., Conley-Lacomb M.K., Chinni S.R. (2012). TMPRSS2-ERG Fusion Gene Expression in Prostate Tumor Cells and Its Clinical and Biological Significance in Prostate Cancer Progression. J. Cancer Sci. Ther..

[37] Kumar-Sinha C., Tomlins S.A., Chinnaiyan A.M. (2008). Recurrent gene fusions in prostate cancer. Nat. Rev. Cancer.

[38] Tomlins S.A., Rhodes D.R., Perner S., Dhanasekaran S.M., Mehra R., Sun X.W., Varambally S., Cao X., Tchinda J., Kuefer R. (2005). Recurrent fusion of TMPRSS2 and ETS transcription factor genes in prostate cancer. Science.

[39] Shang Z., Wang X., Yan H., Cui B., Wang Q., Wu J., Cui X., Li J., Ou T., Yang K. (2018). Intake of Non-steroidal Anti-inflammatory Drugs and the Risk of Prostate Cancer: A Meta-Analysis. Front. Oncol..

[40] Srinivas S., Feldman D. (2009). A phase II trial of calcitriol and naproxen in recurrent prostate cancer. Anticancer Res..

[41] Haffner M.C., Aryee M.J., Toubaji A., Esopi D.M., Albadine R., Gurel B., Isaacs W.B., Bova G.S., Liu W., Xu J. (2010). Androgen-induced TOP2B-mediated double-strand breaks and prostate cancer gene rearrangements. Nat. Genet..

[42] Lin C., Yang L., Tanasa B., Hutt K., Ju B.G., Ohgi K., Zhang J., Rose D.W., Fu X.D., Glass C.K. (2009). Nuclear receptor-induced chromosomal proximity and DNA breaks underlie specific translocations in cancer. Cell.

[43] Mani R.S., Amin M.A., Li X., Kalyana-Sundaram S., Veeneman B.A., Wang L., Ghosh A., Aslam A., Ramanand S.G., Rabquer B.J. (2016). Inflammation-Induced Oxidative Stress Mediates Gene Fusion Formation in Prostate Cancer. Cell Rep..

