# Supplementary material for: Differential Effect of Non-Steroidal Anti-Inflammatory Drugs Aspirin and Naproxen against TMPRSS2-ERG (Fusion)-Driven and Non-Fusion-Driven Prostate Cancer
Source: Cancers (Basel). 2023 Oct 19;15(20):5054. doi: 10.3390/cancers15205054 (PMC10605633; doi:10.3390/cancers15205054)
Supplement: Supplementary file 1 [file cancers-15-05054-s001.zip › cancers-2658067-supplementary.pdf]

Supplementary Materials: Differential Effect of Non-Steroidal Anti-Inflammatory Drugs Aspirin and Naproxen against *TMPRSS2-ERG* (Fusion)-Driven and Non-Fusion-Driven Prostate Cancer

Komal Raina, Kushal Kandhari, Rama Kant, Ram Raj Prasad, Neha Mishra, Akhilendra K. Maurya, Jennifer T. Fox, Shizuko Sei, Robert H. Shoemaker, Maarten C. Bosland, Paul Maroni, Chapla Agarwal and Rajesh Agarwal

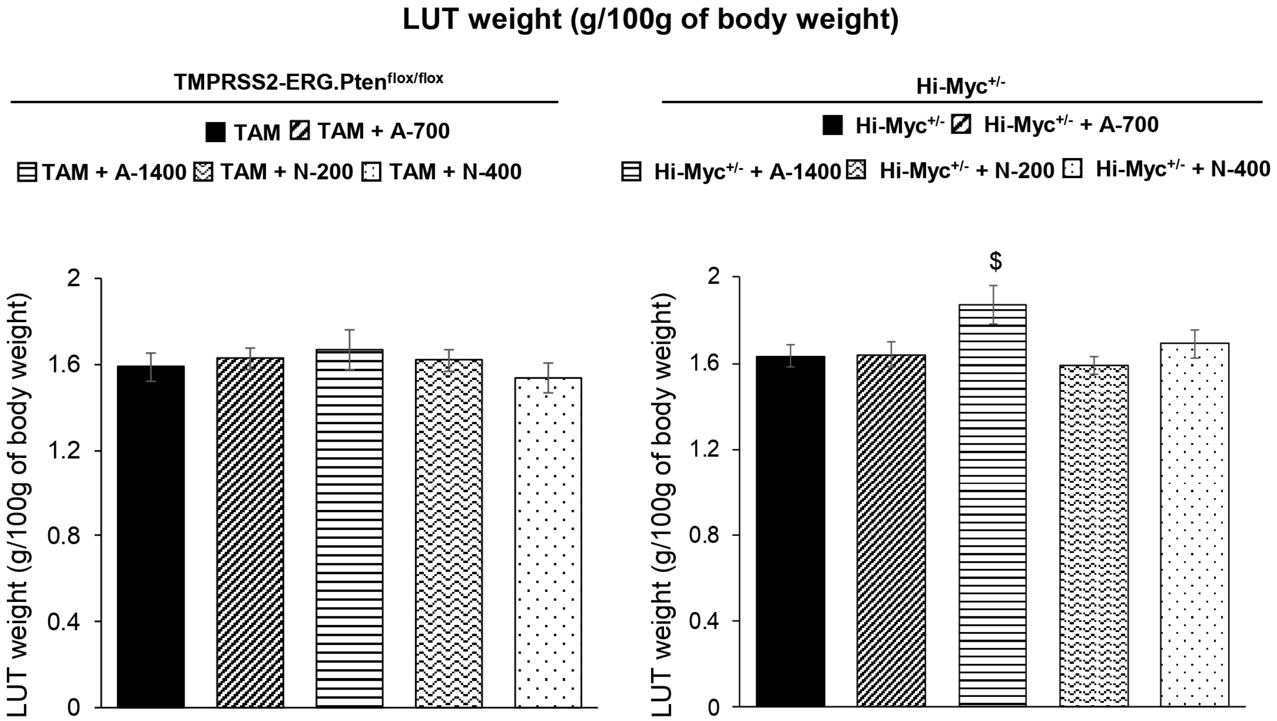

Figure S1. Effect of aspirin and naproxen intervention on the lower urogenital tract (LUT) weight (normalized: g/100g of body weight) of T*MPRSS2-ERG. Pten*$^{flox/flox}$ (+TAM) (*left panel*), and Hi-Myc$^{+/-}$ mice (*right panel*) after aspirin and naproxen intervention. Doses used were aspirin 700ppm (A700); aspirin 1400ppm (A1400); naproxen 200ppm (N200); naproxen 400ppm (N400). In the *TMPRSS2-ERG. Pten*$^{flox/flox}$ efficacy study, 1 week post-Cre-induction (tamoxifen-induced at 8 weeks of mice age) mice were initiated on an NSAID supplemented AIN-76A powder diet until study end (20 weeks post Cre-induction or 28 weeks of mice age). In the Hi-Myc$^{+/-}$ efficacy study, the NSAID-supplemented diets were fed from 6 weeks until 32 weeks of mice age (study end). At the study end, the lower urogenital tract (LUT), including the bladder, seminal vesicles, and prostate, were removed *en bloc*, and LUT wet weight was recorded. Quantified data are represented as Columns (mean for each group): bars represent SEM.**P*≤*0.05.*

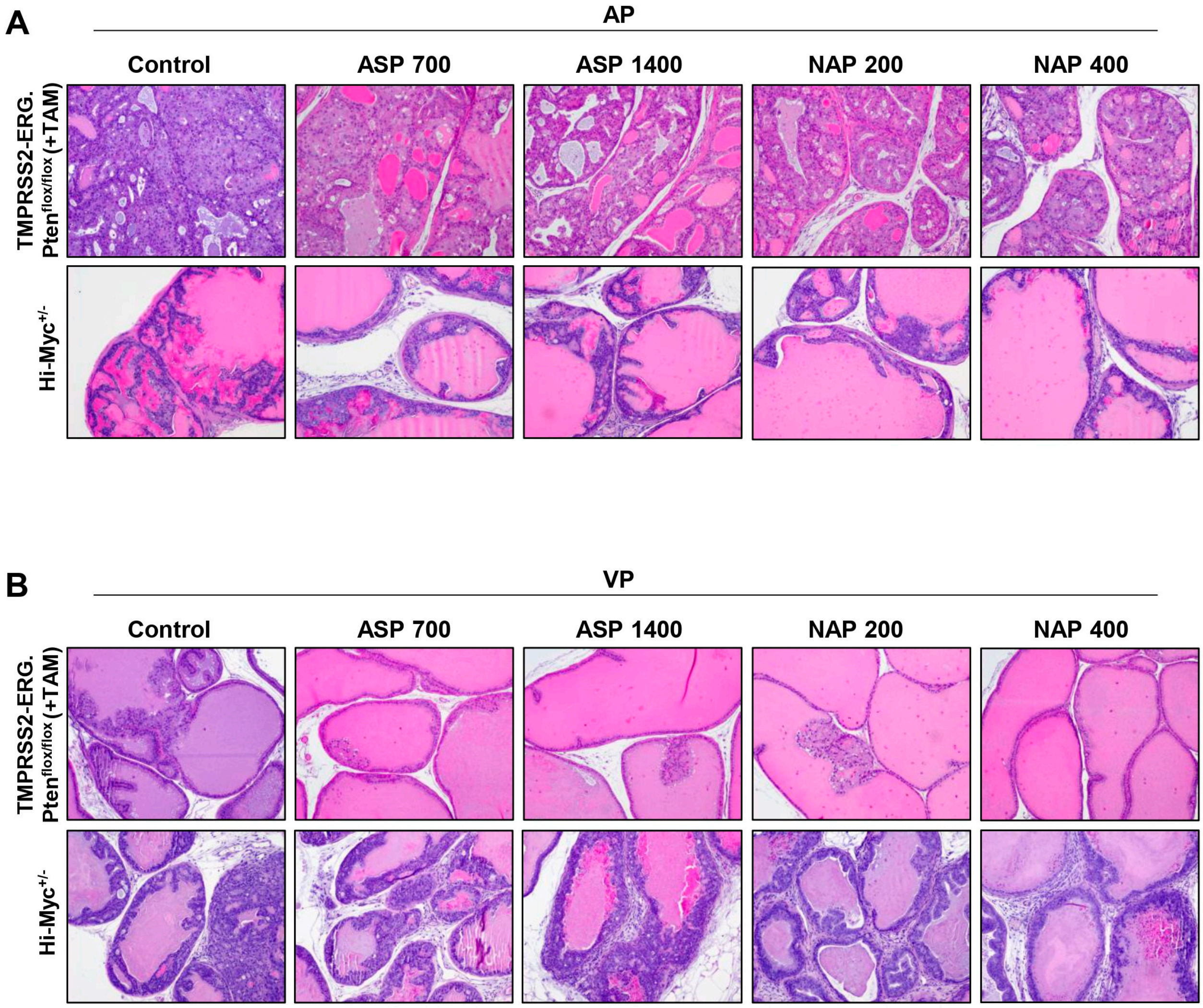

Figure S2. Representative pictographs (x100, H&E images) depicting representative histopathological changes in the anterior and ventral prostate of T*MPRSS2-ERG. Pten*$^{flox/flox}$ (+TAM) and Hi-Myc$^{+/-}$ mice after aspirin and naproxen intervention. Doses used were aspirin 700ppm (A700); aspirin 1400ppm (A1400); naproxen 200ppm (N200); naproxen 400ppm (N400). In the *TMPRSS2-ERG. Pten*$^{flox/flox}$ efficacy study, 1 week post-Cre-induction (tamoxifen-induced at 8 weeks of mice age) mice were initiated on an NSAID supplemented AIN-76A powder diet until study end (20 weeks post Cre-induction or 28 weeks of mice age). In the Hi-Myc$^{+/-}$ efficacy study, the NSAID-supplemented diets were fed from 6 weeks until 32 weeks of mice age (study end).

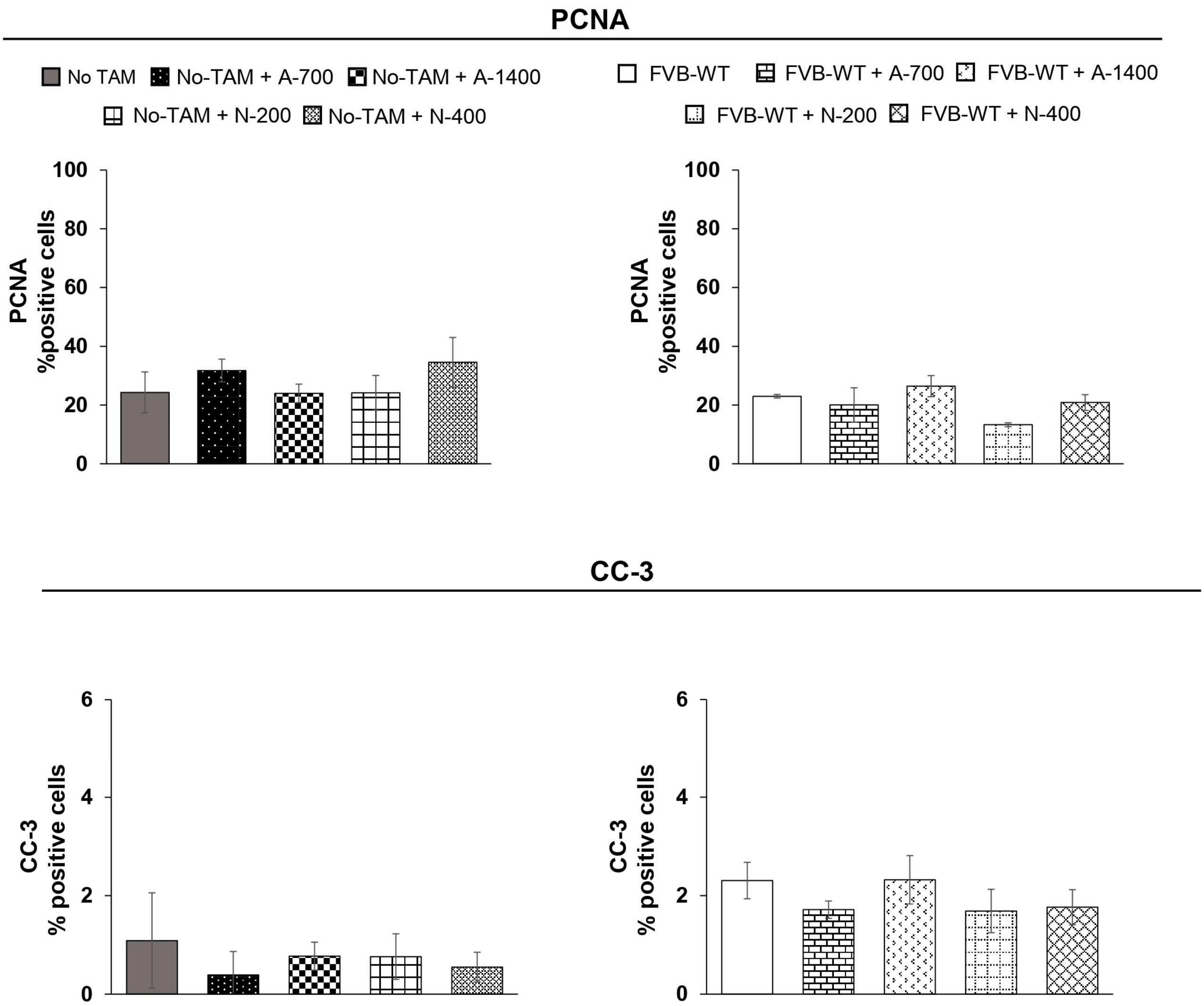
PCNA
No TAM
No-TAM + A-700
No-TAM + A-1400
No-TAM + N-200
No-TAM + N-400
FVB-WT
FVB-WT + A-700
FVB-WT + A-1400
FVB-WT + N-200
FVB-WT + N-400
PCNA %positive cells
100
80
60
40
20
0
CC-3
CC-3 % positive cells
6
4
2
0

Figure S3

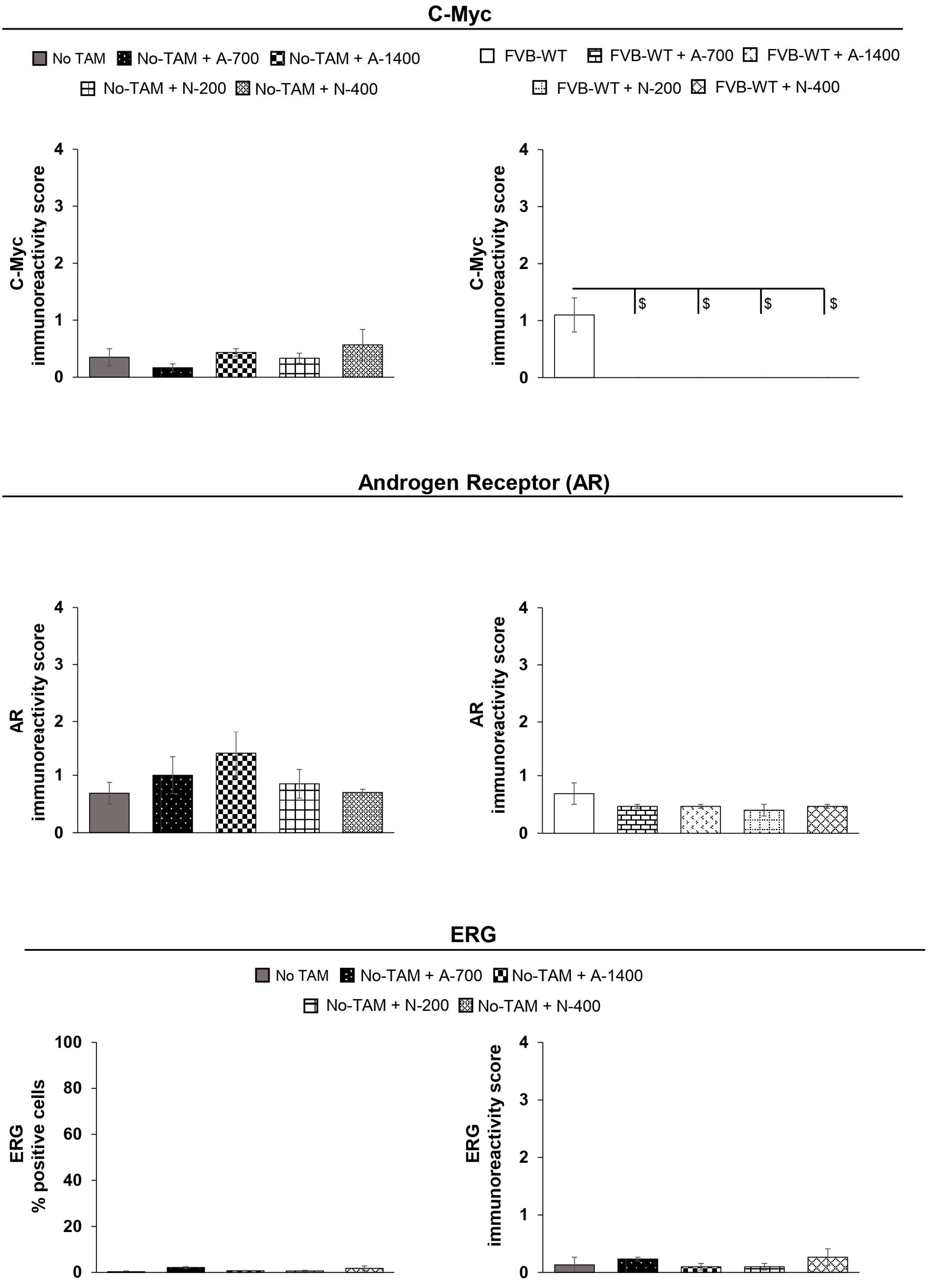
C-Myc
No TAM
No-TAM + A-700
No-TAM + A-1400
No-TAM + N-200
No-TAM + N-400
FVB-WT
FVB-WT + A-700
FVB-WT + A-1400
FVB-WT + N-200
FVB-WT + N-400
C-Myc immunoreactivity score
$
Androgen Receptor (AR)
AR immunoreactivity score
ERG
ERG % positive cells
ERG immunoreactivity score

Figure S4

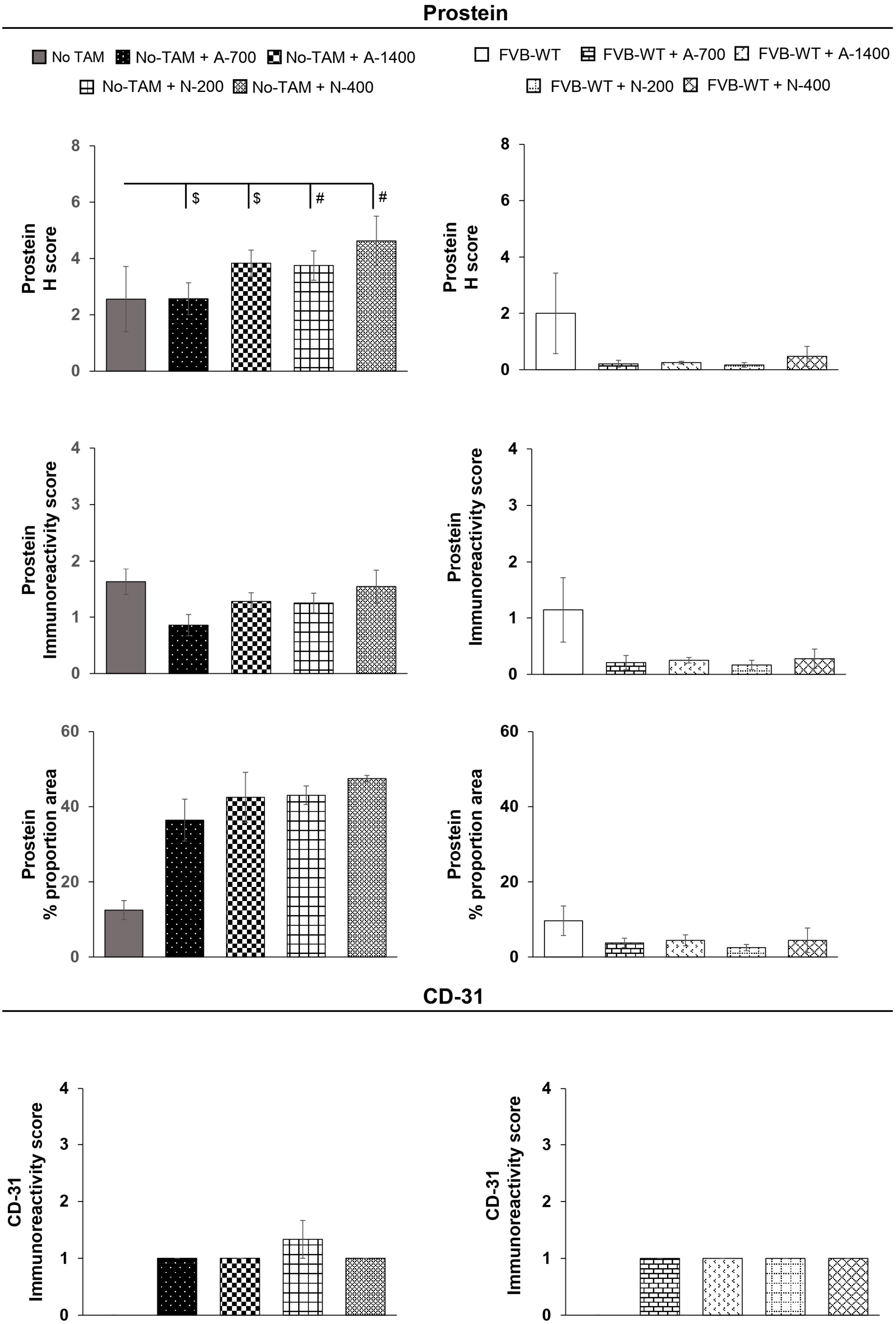

Prostein
No TAM
No-TAM + A-700
No-TAM + A-1400
No-TAM + N-200
No-TAM + N-400
FVB-WT
FVB-WT + A-700
FVB-WT + A-1400
FVB-WT + N-200
FVB-WT + N-400
Prostein H score
$
$
#
#
Prostein Immunoreactivity score
Prostein % proportion area
CD-31
CD-31 Immunoreactivity score

Figure S5

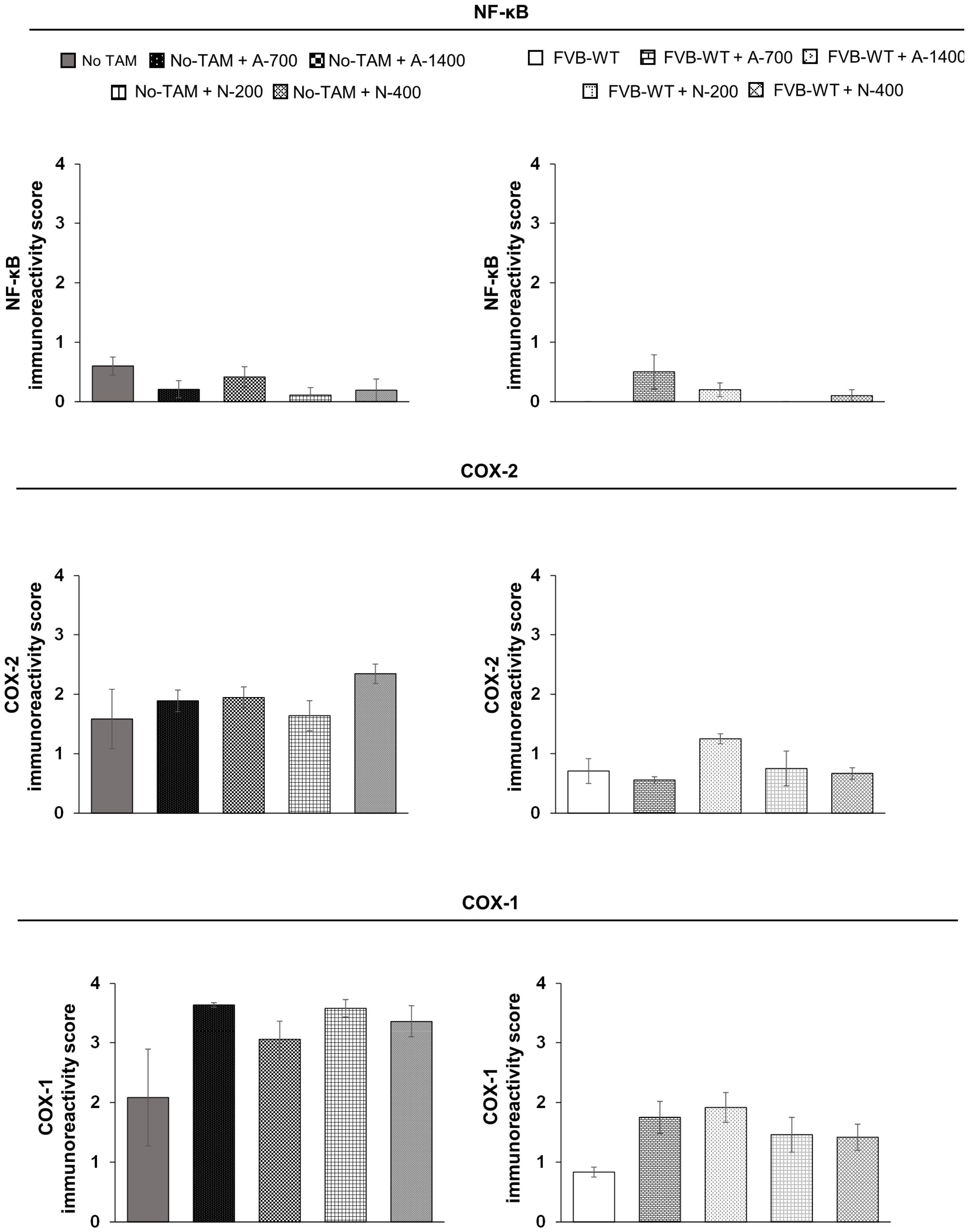

Figure S6

Figure S3–S6. NSAID-feeding effects on PCNA, cleaved caspase-3, c-Myc, Androgen receptor (AR), ERG, prostein (SLC45A3), CD-31 (PECAM-1), NFκB (total p65), COX-2, and COX-1 expression in the dorsolateral prostate of *TMPRSS2-ERG. Pten$^{flox/flox}$* (No-TAM) (*left panel*) and FVB(WT) (*right panel*) mice. In the *TMPRSS2-ERG. Pten$^{flox/flox}$* (No-TAM) mice, the NSAID-supplemented-AIN-76A powder diets were fed from 8 weeks until 28 weeks of mice age. In FVB (WT) mice, the NSAID-supplemented-AIN-76A powder diets were fed from 6 weeks until 32 weeks of mice age (study end). Doses used were aspirin 700ppm (A700); aspirin 1400ppm (A1400); naproxen 200ppm (N200);

naproxen 400ppm (N400). Positive cells were quantified by counting brown-stained cells among the total number of cells in 5-8 randomly selected fields at x400 magnification and plotted as % positive cells. Immunoreactivity (represented by the intensity of brown staining) was scored as 0 (no staining), +1 (weak), +2 (moderate), +3 (strong), and +4 (very strong). H-Score is calculated as [% proportion area of the prostate (positive for prostein expression) x immunoreactivity score (prostein-perinuclear intensity)]. For H-score calculations, the % proportion areas are given arbitrary scores (<1% = 0, ≤ 10% = 1, ≤ 25% = 2, ≤ 50% = 3, ≤ 100 = 4). *, *P≤0.001*; #, *P≤0.01*; $, *P≤0.05.*

Table S1. Experimental groups to determine the preventive efficacy of aspirin and naproxen in the *TMPRSS2-ERG* fusion driven and non-fusion driven models of prostate cancer (PCa).

	Chemopreventive Agent	Dose in AIN-76A powder diet	**Number of Mice per PCa model and Control Groups**			
			**Tamoxifen-induced (+TAM) TMPRSS2-ERG. Pten$^{flox/flox}$**	**Hi-Myc$^{+/-}$**	**Age-matched male controls**	
					**No-Tamoxifen TMPRSS2-ERG. Pten$^{flox/flox}$ mice**	**FVB (WT)**
1	Control diet	0 ppm	25	25	7	7
2	Aspirin	700 ppm	25	25	7	7
3	Aspirin	1400 ppm	25	25	7	7
4	Naproxen	200 ppm	25	25	7	7
5	Naproxen	400 ppm	25	25	7	7
	Total mice		125	125	35	35

Note: Given that at times microdissection of specific lobes was not feasible or if samples were directly snap frozen, for histopathological analysis and immunohistochemistry the number of prostate tissues (from different lobes) assessed were as follows:

**Treatment groups**	**Sample size for histopathology assessment**
**TMPRSS2-ERG. Pten$^{flox/flox}$ (+TAM mice)**	
No-drug controls	DLP (n=21); VP (n=19); AP (n=19)
Aspirin 700 ppm	DLP (n=21); VP (n=19); AP (n=21)
Aspirin 1400 ppm	DLP (n=25); VP (n=20); AP (n=27)
Naproxen 200 ppm	DLP (n=22); VP (n=20); AP (n=24)
Naproxen 400 ppm	DLP (n=29); VP (n=25); AP (n=29)
**Hi-Myc$^{+/-}$ mice**	
No-drug controls	DLP (n=25); VP (n=23); AP (n=26)
Aspirin 700 ppm	DLP (n=24); VP (n=24); AP (n=25)
Aspirin 1400 ppm	DLP (n=21); VP (n=24); AP (n=25)
Naproxen 200 ppm	DLP (n=21); VP (n=19); AP (n=25)
Naproxen 400 ppm	DLP (n=25); VP (n=22); AP (n=25)
	**Sample size for immunohistochemistry**
**TMPRSS2-ERG. Pten$^{flox/flox}$ (+TAM mice)**	Untreated and NSAID-fed: (n=10 tissues/group)

**Hi-Myc$^{+/-}$ mice**	Untreated and NSAID-fed: (n=10 tissues/group)
**TMPRSS2-ERG. Pten$^{flox/flox}$ (No TAM)**	n=4 tissues/group
**FVB (WT)**	n=3 tissues/group

ns = P > 0.05; * = P ≤ 0.05; ** = P ≤ 0.01; *** = P ≤ 0.001.

Table S2. TMPRSS2.ERG. Pten $^{flox/flox}$ (+TAM).

%Area of different tumor grades → DLP: +TAM (28 Weeks age)

**Number of families**	**1**				
**Number of comparisons per family**	21				
**Alpha**	0.05				
**Tukey's multiple comparisons test**	Mean Diff.	95.00% CI of diff.	Significant?	Summary	Adjusted P Value
**Normal vs. LGPIN**	0.000	-11.88 to 11.88	No	ns	>0.9999
**Normal vs. HGPIN**	-62.43	-74.31 to -50.55	Yes	***	<0.0001
**Normal vs. MI**	-5.905	-17.78 to 5.973	No	ns	0.7519
**Normal vs. WD**	-18.19	-30.07 to -6.313	Yes	***	0.0002
**Normal vs. MD**	-13.00	-24.88 to -1.123	Yes	*	0.0221
**Normal vs. PD**	0.000	-11.88 to 11.88	No	ns	>0.9999
**LGPIN vs. HGPIN**	-62.43	-74.31 to -50.55	Yes	***	<0.0001
**LGPIN vs. MI**	-5.905	-17.78 to 5.973	No	ns	0.7519
**LGPIN vs. WD**	-18.19	-30.07 to -6.313	Yes	***	0.0002
**LGPIN vs. MD**	-13.00	-24.88 to -1.123	Yes	*	0.0221
**LGPIN vs. PD**	0.000	-11.88 to 11.88	No	ns	>0.9999
**HGPIN vs. MI**	56.52	44.65 to 68.40	Yes	***	<0.0001
**HGPIN vs. WD**	44.24	32.36 to 56.12	Yes	***	<0.0001
**HGPIN vs. MD**	49.43	37.55 to 61.31	Yes	***	<0.0001
**HGPIN vs. PD**	62.43	50.55 to 74.31	Yes	***	<0.0001
**MI vs. WD**	-12.29	-24.16 to -0.4083	Yes	*	0.0375
**MI vs. MD**	-7.095	-18.97 to 4.782	No	ns	0.5589
**MI vs. PD**	5.905	-5.973 to 17.78	No	ns	0.7519
**WD vs. MD**	5.190	-6.687 to 17.07	No	ns	0.8475
**WD vs. PD**	18.19	6.313 to 30.07	Yes	***	0.0002
**MD vs. PD**	13.00	1.123 to 24.88	Yes	*	0.0221

%Area of different tumor grades → DLP: TAM + ASP 700 (28 Weeks age)

Number of families	1				
Number of comparisons per family	21				
Alpha	0.05				
Tukey's multiple comparisons test	Mean Diff.	95.00% CI of diff.	Significant?	Summary	Adjusted P Value
Normal vs. LGPIN	-23.81	-35.96 to -11.66	Yes	***	<0.0001
Normal vs. HGPIN	-67.95	-80.10 to -55.80	Yes	***	<0.0001
Normal vs. MI	-3.952	-16.10 to 8.196	No	ns	0.9589
Normal vs. WD	-3.810	-15.96 to 8.339	No	ns	0.9657
Normal vs. MD	-0.4762	-12.62 to 11.67	No	ns	>0.9999
Normal vs. PD	0.000	-12.15 to 12.15	No	ns	>0.9999
LGPIN vs. HGPIN	-44.14	-56.29 to -31.99	Yes	***	<0.0001
LGPIN vs. MI	19.86	7.709 to 32.01	Yes	***	<0.0001
LGPIN vs. WD	20.00	7.852 to 32.15	Yes	***	<0.0001
LGPIN vs. MD	23.33	11.18 to 35.48	Yes	***	<0.0001
LGPIN vs. PD	23.81	11.66 to 35.96	Yes	***	<0.0001
HGPIN vs. MI	64.00	51.85 to 76.15	Yes	***	<0.0001
HGPIN vs. WD	64.14	51.99 to 76.29	Yes	***	<0.0001
HGPIN vs. MD	67.48	55.33 to 79.62	Yes	***	<0.0001
HGPIN vs. PD	67.95	55.80 to 80.10	Yes	***	<0.0001
MI vs. WD	0.1429	-12.01 to 12.29	No	ns	>0.9999
MI vs. MD	3.476	-8.672 to 15.62	No	ns	0.9783
MI vs. PD	3.952	-8.196 to 16.10	No	ns	0.9589
WD vs. MD	3.333	-8.815 to 15.48	No	ns	0.9825
WD vs. PD	3.810	-8.339 to 15.96	No	ns	0.9657
MD vs. PD	0.4762	-11.67 to 12.62	No	ns	>0.9999

%Area of different tumor grades → DLP: TAM + ASP 1400 (28 Weeks age)

Number of families	1				
Number of comparisons per family	21				
Alpha	0.05				
Tukey's multiple comparisons test	Mean Diff.	95.00% CI of diff.	Significant?	Summary	Adjusted P Value
Normal vs. LGPIN	-12.20	-24.15 to -0.2543	Yes	*	0.0419
Normal vs. HGPIN	-69.92	-81.87 to -57.97	Yes	***	<0.0001
Normal vs. MI	-4.800	-16.75 to 7.146	No	ns	0.8937
Normal vs. WD	-10.68	-22.63 to 1.266	No	ns	0.1130

**Normal vs. MD**	-2.400	-14.35 to 9.546	No	ns	0.9968
**Normal vs. PD**	0.000	-11.95 to 11.95	No	ns	>0.9999
**LGPIN vs. HGPIN**	-57.72	-69.67 to -45.77	Yes	***	<0.0001
**LGPIN vs. MI**	7.400	-4.546 to 19.35	No	ns	0.5173
**LGPIN vs. WD**	1.520	-10.43 to 13.47	No	ns	0.9998
**LGPIN vs. MD**	9.800	-2.146 to 21.75	No	ns	0.1854
**LGPIN vs. PD**	12.20	0.2543 to 24.15	Yes	*	0.0419
**HGPIN vs. MI**	65.12	53.17 to 77.07	Yes	***	<0.0001
**HGPIN vs. WD**	59.24	47.29 to 71.19	Yes	***	<0.0001
**HGPIN vs. MD**	67.52	55.57 to 79.47	Yes	***	<0.0001
**HGPIN vs. PD**	69.92	57.97 to 81.87	Yes	***	<0.0001
**MI vs. WD**	-5.880	-17.83 to 6.066	No	ns	0.7627
**MI vs. MD**	2.400	-9.546 to 14.35	No	ns	0.9968
**MI vs. PD**	4.800	-7.146 to 16.75	No	ns	0.8937
**WD vs. MD**	8.280	-3.666 to 20.23	No	ns	0.3762
**WD vs. PD**	10.68	-1.266 to 22.63	No	ns	0.1130
**MD vs. PD**	2.400	-9.546 to 14.35	No	ns	0.9968

%Area of different tumor grades → DLP: TAM + NAP 200 (28 Weeks age)

**Number of families**	**1**				
**Number of comparisons per family**	21				
**Alpha**	0.05				
**Tukey's multiple comparisons test**	Mean Diff.	95.00% CI of diff.	Significant?	Summary	Adjusted P Value
**Normal vs. LGPIN**	-34.66	-47.54 to -21.79	Yes	***	<0.0001
**Normal vs. HGPIN**	-61.74	-74.62 to -48.87	Yes	***	<0.0001
**Normal vs. MI**	-2.682	-15.56 to 10.19	No	ns	0.9960
**Normal vs. WD**	-0.9091	-13.78 to 11.97	No	ns	>0.9999
**Normal vs. MD**	0.000	-12.88 to 12.88	No	ns	>0.9999
**Normal vs. PD**	0.000	-12.88 to 12.88	No	ns	>0.9999
**LGPIN vs. HGPIN**	-27.08	-39.95 to -14.20	Yes	***	<0.0001
**LGPIN vs. MI**	31.98	19.11 to 44.86	Yes	***	<0.0001
**LGPIN vs. WD**	33.75	20.88 to 46.63	Yes	***	<0.0001
**LGPIN vs. MD**	34.66	21.79 to 47.54	Yes	***	<0.0001
**LGPIN vs. PD**	34.66	21.79 to 47.54	Yes	***	<0.0001
**HGPIN vs. MI**	59.06	46.18 to 71.94	Yes	***	<0.0001
**HGPIN vs. WD**	60.83	47.96 to 73.71	Yes	***	<0.0001
**HGPIN vs. MD**	61.74	48.87 to 74.62	Yes	***	<0.0001
**HGPIN vs. PD**	61.74	48.87 to 74.62	Yes	***	<0.0001

**MI vs. WD**	1.773	-11.10 to 14.65	No	ns	0.9996
**MI vs. MD**	2.682	-10.19 to 15.56	No	ns	0.9960
**MI vs. PD**	2.682	-10.19 to 15.56	No	ns	0.9960
**WD vs. MD**	0.9091	-11.97 to 13.78	No	ns	>0.9999
**WD vs. PD**	0.9091	-11.97 to 13.78	No	ns	>0.9999
**MD vs. PD**	0.000	-12.88 to 12.88	No	ns	>0.9999

%Area of different tumor grades → DLP: TAM + NAP 400 (28 Weeks age)

**Number of families**	**1**				
**Number of comparisons per family**	21				
**Alpha**	0.05				
**Tukey's multiple comparisons test**	Mean Diff.	95.00% CI of diff.	Significant?	Summary	Adjusted P Value
**Normal vs. LGPIN**	-34.31	-46.01 to -22.61	Yes	***	<0.0001
**Normal vs. HGPIN**	-61.07	-72.77 to -49.37	Yes	***	<0.0001
**Normal vs. MI**	-3.759	-15.46 to 7.944	No	ns	0.9624
**Normal vs. WD**	-0.8621	-12.56 to 10.84	No	ns	>0.9999
**Normal vs. MD**	0.000	-11.70 to 11.70	No	ns	>0.9999
**Normal vs. PD**	0.000	-11.70 to 11.70	No	ns	>0.9999
**LGPIN vs. HGPIN**	-26.76	-38.46 to -15.06	Yes	***	<0.0001
**LGPIN vs. MI**	30.55	18.85 to 42.25	Yes	***	<0.0001
**LGPIN vs. WD**	33.45	21.75 to 45.15	Yes	***	<0.0001
**LGPIN vs. MD**	34.31	22.61 to 46.01	Yes	***	<0.0001
**LGPIN vs. PD**	34.31	22.61 to 46.01	Yes	***	<0.0001
**HGPIN vs. MI**	57.31	45.61 to 69.01	Yes	***	<0.0001
**HGPIN vs. WD**	60.21	48.50 to 71.91	Yes	***	<0.0001
**HGPIN vs. MD**	61.07	49.37 to 72.77	Yes	***	<0.0001
**HGPIN vs. PD**	61.07	49.37 to 72.77	Yes	***	<0.0001
**MI vs. WD**	2.897	-8.806 to 14.60	No	ns	0.9901
**MI vs. MD**	3.759	-7.944 to 15.46	No	ns	0.9624
**MI vs. PD**	3.759	-7.944 to 15.46	No	ns	0.9624
**WD vs. MD**	0.8621	-10.84 to 12.56	No	ns	>0.9999
**WD vs. PD**	0.8621	-10.84 to 12.56	No	ns	>0.9999
**MD vs. PD**	0.000	-11.70 to 11.70	No	ns	>0.9999

%Area of different tumor grades → VP: +TAM (28 weeks age)

**Number of families**	**1**				
**Number of comparisons per family**	21				
**Alpha**	0.05				
**Tukey's multiple comparisons test**	Mean Diff.	95.00% CI of diff.	Significant?	Summary	Adjusted P Value
**Normal vs. LGPIN**	-90.53	-103.2 to -77.86	Yes	***	<0.0001
**Normal vs. HGPIN**	-9.474	-22.14 to 3.193	No	ns	0.2814
**Normal vs. MI**	0.000	-12.67 to 12.67	No	ns	>0.9999
**Normal vs. WD**	0.000	-12.67 to 12.67	No	ns	>0.9999
**Normal vs. MD**	0.000	-12.67 to 12.67	No	ns	>0.9999
**Normal vs. PD**	0.000	-12.67 to 12.67	No	ns	>0.9999
**LGPIN vs. HGPIN**	81.05	68.39 to 93.72	Yes	***	<0.0001
**LGPIN vs. MI**	90.53	77.86 to 103.2	Yes	***	<0.0001
**LGPIN vs. WD**	90.53	77.86 to 103.2	Yes	***	<0.0001
**LGPIN vs. MD**	90.53	77.86 to 103.2	Yes	***	<0.0001
**LGPIN vs. PD**	90.53	77.86 to 103.2	Yes	***	<0.0001
**HGPIN vs. MI**	9.474	-3.193 to 22.14	No	ns	0.2814
**HGPIN vs. WD**	9.474	-3.193 to 22.14	No	ns	0.2814
**HGPIN vs. MD**	9.474	-3.193 to 22.14	No	ns	0.2814
**HGPIN vs. PD**	9.474	-3.193 to 22.14	No	ns	0.2814
**MI vs. WD**	0.000	-12.67 to 12.67	No	ns	>0.9999
**MI vs. MD**	0.000	-12.67 to 12.67	No	ns	>0.9999
**MI vs. PD**	0.000	-12.67 to 12.67	No	ns	>0.9999
**WD vs. MD**	0.000	-12.67 to 12.67	No	ns	>0.9999
**WD vs. PD**	0.000	-12.67 to 12.67	No	ns	>0.9999
**MD vs. PD**	0.000	-12.67 to 12.67	No	ns	>0.9999

%Area of different tumor grades → VP: TAM + ASP 700 (28 Weeks age)

**Number of families**	**1**				
**Number of comparisons per family**	21				
**Alpha**	0.05				
**Tukey's multiple comparisons test**	Mean Diff.	95.00% CI of diff.	Significant?	Summary	Adjusted P Value
**Normal vs. LGPIN**	-98.95	-100.3 to -97.56	Yes	***	<0.0001
**Normal vs. HGPIN**	-1.053	-2.444 to 0.3383	No	ns	0.2680
**Normal vs. MI**	0.000	-1.391 to 1.391	No	ns	>0.9999
**Normal vs. WD**	0.000	-1.391 to 1.391	No	ns	>0.9999

**Normal vs. MD**	0.000	-1.391 to 1.391	No	ns	>0.9999
**Normal vs. PD**	0.000	-1.391 to 1.391	No	ns	>0.9999
**LGPIN vs. HGPIN**	97.89	96.50 to 99.29	Yes	***	<0.0001
**LGPIN vs. MI**	98.95	97.56 to 100.3	Yes	***	<0.0001
**LGPIN vs. WD**	98.95	97.56 to 100.3	Yes	***	<0.0001
**LGPIN vs. MD**	98.95	97.56 to 100.3	Yes	***	<0.0001
**LGPIN vs. PD**	98.95	97.56 to 100.3	Yes	***	<0.0001
**HGPIN vs. MI**	1.053	-0.3383 to 2.444	No	ns	0.2680
**HGPIN vs. WD**	1.053	-0.3383 to 2.444	No	ns	0.2680
**HGPIN vs. MD**	1.053	-0.3383 to 2.444	No	ns	0.2680
**HGPIN vs. PD**	1.053	-0.3383 to 2.444	No	ns	0.2680
**MI vs. WD**	0.000	-1.391 to 1.391	No	ns	>0.9999
**MI vs. MD**	0.000	-1.391 to 1.391	No	ns	>0.9999
**MI vs. PD**	0.000	-1.391 to 1.391	No	ns	>0.9999
**WD vs. MD**	0.000	-1.391 to 1.391	No	ns	>0.9999
**WD vs. PD**	0.000	-1.391 to 1.391	No	ns	>0.9999
**MD vs. PD**	0.000	-1.391 to 1.391	No	ns	>0.9999

%Area of different tumor grades → VP: TAM + ASP 1400 (28 Weeks age)

**Number of families**	**1**				
**Number of comparisons per family**	21				
**Alpha**	0.05				
**Tukey's multiple comparisons test**	Mean Diff.	95.00% CI of diff.	Significant?	Summary	Adjusted P Value
**Normal vs. LGPIN**	-78.50	-96.22 to -60.78	Yes	***	<0.0001
**Normal vs. HGPIN**	-9.000	-26.72 to 8.716	No	ns	0.7318
**Normal vs. MI**	3.500	-14.22 to 21.22	No	ns	0.9970
**Normal vs. WD**	4.000	-13.72 to 21.72	No	ns	0.9937
**Normal vs. MD**	4.000	-13.72 to 21.72	No	ns	0.9937
**Normal vs. PD**	4.000	-13.72 to 21.72	No	ns	0.9937
**LGPIN vs. HGPIN**	69.50	51.78 to 87.22	Yes	***	<0.0001
**LGPIN vs. MI**	82.00	64.28 to 99.72	Yes	***	<0.0001
**LGPIN vs. WD**	82.50	64.78 to 100.2	Yes	***	<0.0001
**LGPIN vs. MD**	82.50	64.78 to 100.2	Yes	***	<0.0001
**LGPIN vs. PD**	82.50	64.78 to 100.2	Yes	***	<0.0001
**HGPIN vs. MI**	12.50	-5.216 to 30.22	No	ns	0.3515
**HGPIN vs. WD**	13.00	-4.716 to 30.72	No	ns	0.3042
**HGPIN vs. MD**	13.00	-4.716 to 30.72	No	ns	0.3042
**HGPIN vs. PD**	13.00	-4.716 to 30.72	No	ns	0.3042

**MI vs. WD**	0.5000	-17.22 to 18.22	No	ns	>0.9999
**MI vs. MD**	0.5000	-17.22 to 18.22	No	ns	>0.9999
**MI vs. PD**	0.5000	-17.22 to 18.22	No	ns	>0.9999
**WD vs. MD**	0.000	-17.72 to 17.72	No	ns	>0.9999
**WD vs. PD**	0.000	-17.72 to 17.72	No	ns	>0.9999
**MD vs. PD**	0.000	-17.72 to 17.72	No	ns	>0.9999

%Area of different tumor grades → VP: TAM + NAP 200 (28 Weeks age)

**Number of families**	**1**				
**Number of comparisons per family**	21				
**Alpha**	0.05				
**Tukey's multiple comparisons test**	Mean Diff.	95.00% CI of diff.	Significant?	Summary	Adjusted P Value
**Normal vs. LGPIN**	-73.50	-84.69 to -62.31	Yes	***	<0.0001
**Normal vs. HGPIN**	9.500	-1.693 to 20.69	No	ns	0.1534
**Normal vs. MI**	12.00	0.8066 to 23.19	Yes	*	0.0271
**Normal vs. WD**	12.00	0.8066 to 23.19	Yes	*	0.0271
**Normal vs. MD**	12.00	0.8066 to 23.19	Yes	*	0.0271
**Normal vs. PD**	12.00	0.8066 to 23.19	Yes	*	0.0271
**LGPIN vs. HGPIN**	83.00	71.81 to 94.19	Yes	***	<0.0001
**LGPIN vs. MI**	85.50	74.31 to 96.69	Yes	***	<0.0001
**LGPIN vs. WD**	85.50	74.31 to 96.69	Yes	***	<0.0001
**LGPIN vs. MD**	85.50	74.31 to 96.69	Yes	***	<0.0001
**LGPIN vs. PD**	85.50	74.31 to 96.69	Yes	***	<0.0001
**HGPIN vs. MI**	2.500	-8.693 to 13.69	No	ns	0.9941
**HGPIN vs. WD**	2.500	-8.693 to 13.69	No	ns	0.9941
**HGPIN vs. MD**	2.500	-8.693 to 13.69	No	ns	0.9941
**HGPIN vs. PD**	2.500	-8.693 to 13.69	No	ns	0.9941
**MI vs. WD**	0.000	-11.19 to 11.19	No	ns	>0.9999
**MI vs. MD**	0.000	-11.19 to 11.19	No	ns	>0.9999
**MI vs. PD**	0.000	-11.19 to 11.19	No	ns	>0.9999
**WD vs. MD**	0.000	-11.19 to 11.19	No	ns	>0.9999
**WD vs. PD**	0.000	-11.19 to 11.19	No	ns	>0.9999
**MD vs. PD**	0.000	-11.19 to 11.19	No	ns	>0.9999

%Area of different tumor grades → VP: TAM + NAP 400 (28 Weeks age)

**Number of families**	**1**				
**Number of comparisons per family**	21				
**Alpha**	0.05				
**Tukey's multiple comparisons test**	Mean Diff.	95.00% CI of diff.	Significant?	Summary	Adjusted P Value
**Normal vs. LGPIN**	-61.20	-76.88 to -45.52	Yes	***	<0.0001
**Normal vs. HGPIN**	12.80	-2.878 to 28.48	No	ns	0.1901
**Normal vs. MI**	17.20	1.522 to 32.88	Yes	*	0.0215
**Normal vs. WD**	17.20	1.522 to 32.88	Yes	*	0.0215
**Normal vs. MD**	17.20	1.522 to 32.88	Yes	*	0.0215
**Normal vs. PD**	17.20	1.522 to 32.88	Yes	*	0.0215
**LGPIN vs. HGPIN**	74.00	58.32 to 89.68	Yes	***	<0.0001
**LGPIN vs. MI**	78.40	62.72 to 94.08	Yes	***	<0.0001
**LGPIN vs. WD**	78.40	62.72 to 94.08	Yes	***	<0.0001
**LGPIN vs. MD**	78.40	62.72 to 94.08	Yes	***	<0.0001
**LGPIN vs. PD**	78.40	62.72 to 94.08	Yes	***	<0.0001
**HGPIN vs. MI**	4.400	-11.28 to 20.08	No	ns	0.9806
**HGPIN vs. WD**	4.400	-11.28 to 20.08	No	ns	0.9806
**HGPIN vs. MD**	4.400	-11.28 to 20.08	No	ns	0.9806
**HGPIN vs. PD**	4.400	-11.28 to 20.08	No	ns	0.9806
**MI vs. WD**	0.000	-15.68 to 15.68	No	ns	>0.9999
**MI vs. MD**	0.000	-15.68 to 15.68	No	ns	>0.9999
**MI vs. PD**	0.000	-15.68 to 15.68	No	ns	>0.9999
**WD vs. MD**	0.000	-15.68 to 15.68	No	ns	>0.9999
**WD vs. PD**	0.000	-15.68 to 15.68	No	ns	>0.9999
**MD vs. PD**	0.000	-15.68 to 15.68	No	ns	>0.9999

%Area of different tumor grades → AP: +TAM (28 Week age)

**Number of families**	**1**				
**Number of comparisons per family**	21				
**Alpha**	0.05				
**Tukey's multiple comparisons test**	Mean Diff.	95.00% CI of diff.	Significant?	Summary	Adjusted P Value
**Normal vs. LGPIN**	-34.58	-60.56 to -8.602	Yes	**	0.0021
**Normal vs. HGPIN**	-38.21	-64.19 to -12.23	Yes	***	0.0004
**Normal vs. MI**	-1.053	-27.03 to 24.92	No	ns	>0.9999

**Normal vs. WD**	-11.53	-37.50 to 14.45	No	ns	0.8368
**Normal vs. MD**	-14.11	-40.08 to 11.87	No	ns	0.6652
**Normal vs. PD**	0.000	-25.98 to 25.98	No	ns	>0.9999
**LGPIN vs. HGPIN**	-3.632	-29.61 to 22.35	No	ns	0.9996
**LGPIN vs. MI**	33.53	7.549 to 59.50	Yes	**	0.0033
**LGPIN vs. WD**	23.05	-2.924 to 49.03	No	ns	0.1176
**LGPIN vs. MD**	20.47	-5.503 to 46.45	No	ns	0.2238
**LGPIN vs. PD**	34.58	8.602 to 60.56	Yes	**	0.0021
**HGPIN vs. MI**	37.16	11.18 to 63.13	Yes	***	0.0007
**HGPIN vs. WD**	26.68	0.7071 to 52.66	Yes	*	0.0399
**HGPIN vs. MD**	24.11	-1.872 to 50.08	No	ns	0.0877
**HGPIN vs. PD**	38.21	12.23 to 64.19	Yes	***	0.0004
**MI vs. WD**	-10.47	-36.45 to 15.50	No	ns	0.8898
**MI vs. MD**	-13.05	-39.03 to 12.92	No	ns	0.7410
**MI vs. PD**	1.053	-24.92 to 27.03	No	ns	>0.9999
**WD vs. MD**	-2.579	-28.56 to 23.40	No	ns	>0.9999
**WD vs. PD**	11.53	-14.45 to 37.50	No	ns	0.8368
**MD vs. PD**	14.11	-11.87 to 40.08	No	ns	0.6652

%Area of different tumor grades → AP: TAM + ASP 700 (28 Week age)

**Number of families**	**1**				
**Number of comparisons per family**	15				
**Alpha**	0.05				
**Tukey's multiple comparisons test**	Mean Diff.	95.00% CI of diff.	Significant?	Summary	Adjusted P Value
**Normal vs. LGPIN**	-31.90	-52.90 to -10.91	Yes	***	0.0003
**Normal vs. HGPIN**	-61.67	-82.66 to -40.68	Yes	***	<0.0001
**Normal vs. WD**	-3.333	-24.32 to 17.66	No	ns	0.9974
**Normal vs. MD**	-3.095	-24.09 to 17.90	No	ns	0.9982
**Normal vs. PD**	0.000	-20.99 to 20.99	No	ns	>0.9999
**LGPIN vs. HGPIN**	-29.76	-50.75 to -8.771	Yes	**	0.0010
**LGPIN vs. WD**	28.57	7.581 to 49.56	Yes	**	0.0019
**LGPIN vs. MD**	28.81	7.819 to 49.80	Yes	**	0.0016
**LGPIN vs. PD**	31.90	10.91 to 52.90	Yes	***	0.0003
**HGPIN vs. WD**	58.33	37.34 to 79.32	Yes	***	<0.0001
**HGPIN vs. MD**	58.57	37.58 to 79.56	Yes	***	<0.0001
**HGPIN vs. PD**	61.67	40.68 to 82.66	Yes	***	<0.0001
**WD vs. MD**	0.2381	-20.75 to 21.23	No	ns	>0.9999
**WD vs. PD**	3.333	-17.66 to 24.32	No	ns	0.9974

**MD vs. PD**	3.095	-17.90 to 24.09	No	ns	0.9982

%Area of different tumor grades → AP: TAM + ASP 1400 (28 Week age)

**Number of families**	**1**				
**Number of comparisons per family**	21				
**Alpha**	0.05				
**Tukey's multiple comparisons test**	Mean Diff.	95.00% CI of diff.	Significant?	Summary	Adjusted P Value
**Normal vs. LGPIN**	-44.44	-62.58 to -26.31	Yes	***	<0.0001
**Normal vs. HGPIN**	-37.78	-55.91 to -19.65	Yes	***	<0.0001
**Normal vs. MI**	-0.9259	-19.06 to 17.21	No	ns	>0.9999
**Normal vs. WD**	-10.19	-28.32 to 7.946	No	ns	0.6336
**Normal vs. MD**	-6.667	-24.80 to 11.46	No	ns	0.9285
**Normal vs. PD**	0.000	-18.13 to 18.13	No	ns	>0.9999
**LGPIN vs. HGPIN**	6.667	-11.46 to 24.80	No	ns	0.9285
**LGPIN vs. MI**	43.52	25.39 to 61.65	Yes	***	<0.0001
**LGPIN vs. WD**	34.26	16.13 to 52.39	Yes	***	<0.0001
**LGPIN vs. MD**	37.78	19.65 to 55.91	Yes	***	<0.0001
**LGPIN vs. PD**	44.44	26.31 to 62.58	Yes	***	<0.0001
**HGPIN vs. MI**	36.85	18.72 to 54.98	Yes	***	<0.0001
**HGPIN vs. WD**	27.59	9.461 to 45.72	Yes	***	0.0002
**HGPIN vs. MD**	31.11	12.98 to 49.24	Yes	***	<0.0001
**HGPIN vs. PD**	37.78	19.65 to 55.91	Yes	***	<0.0001
**MI vs. WD**	-9.259	-27.39 to 8.872	No	ns	0.7309
**MI vs. MD**	-5.741	-23.87 to 12.39	No	ns	0.9648
**MI vs. PD**	0.9259	-17.21 to 19.06	No	ns	>0.9999
**WD vs. MD**	3.519	-14.61 to 21.65	No	ns	0.9973
**WD vs. PD**	10.19	-7.946 to 28.32	No	ns	0.6336
**MD vs. PD**	6.667	-11.46 to 24.80	No	ns	0.9285

%Area of different tumor grades → AP: TAM + NAP 200 (28 Week age)

**Number of families**	**1**				
**Number of comparisons per family**	21				
**Alpha**	0.05				
**Tukey's multiple comparisons test**	Mean Diff.	95.00% CI of diff.	Significant?	Summary	Adjusted P Value

**Normal vs. LGPIN**	-36.04	-60.64 to -11.45	Yes	***	0.0004
**Normal vs. HGPIN**	-26.25	-50.84 to -1.656	Yes	*	0.0282
**Normal vs. MI**	10.42	-76.53 to 97.37	No	ns	0.9998
**Normal vs. WD**	6.042	-18.55 to 30.64	No	ns	0.9901
**Normal vs. MD**	8.333	-16.26 to 32.93	No	ns	0.9501
**Normal vs. PD**	10.42	-14.18 to 35.01	No	ns	0.8656
**LGPIN vs. HGPIN**	9.792	-14.80 to 34.39	No	ns	0.8964
**LGPIN vs. MI**	46.46	-40.49 to 133.4	No	ns	0.6833
**LGPIN vs. WD**	42.08	17.49 to 66.68	Yes	***	<0.0001
**LGPIN vs. MD**	44.38	19.78 to 68.97	Yes	***	<0.0001
**LGPIN vs. PD**	46.46	21.86 to 71.05	Yes	***	<0.0001
**HGPIN vs. MI**	36.67	-50.28 to 123.6	No	ns	0.8680
**HGPIN vs. WD**	32.29	7.698 to 56.89	Yes	**	0.0025
**HGPIN vs. MD**	34.58	9.990 to 59.18	Yes	***	0.0009
**HGPIN vs. PD**	36.67	12.07 to 61.26	Yes	***	0.0003
**MI vs. WD**	-4.375	-91.33 to 82.58	No	ns	>0.9999
**MI vs. MD**	-2.083	-89.03 to 84.87	No	ns	>0.9999
**MI vs. PD**	0.000	-86.95 to 86.95	No	ns	>0.9999
**WD vs. MD**	2.292	-22.30 to 26.89	No	ns	>0.9999
**WD vs. PD**	4.375	-20.22 to 28.97	No	ns	0.9983
**MD vs. PD**	2.083	-22.51 to 26.68	No	ns	>0.9999

%Area of different tumor grades → AP: TAM + NAP 400 (28 Week age)

**Number of families**	**1**				
**Number of comparisons per family**	21				
**Alpha**	0.05				
**Tukey's multiple comparisons test**	Mean Diff.	95.00% CI of diff.	Significant?	Summary	Adjusted P Value
**Normal vs. LGPIN**	-34.07	-53.58 to -14.56	Yes	***	<0.0001
**Normal vs. HGPIN**	-25.45	-44.95 to -5.942	Yes	**	0.0026
**Normal vs. MI**	8.517	-10.99 to 28.02	No	ns	0.8509
**Normal vs. WD**	-3.828	-23.33 to 15.68	No	ns	0.9972
**Normal vs. MD**	6.552	-12.95 to 26.06	No	ns	0.9534
**Normal vs. PD**	8.621	-10.89 to 28.13	No	ns	0.8435
**LGPIN vs. HGPIN**	8.621	-10.89 to 28.13	No	ns	0.8435
**LGPIN vs. MI**	42.59	23.08 to 62.09	Yes	***	<0.0001
**LGPIN vs. WD**	30.24	10.74 to 49.75	Yes	***	0.0001
**LGPIN vs. MD**	40.62	21.11 to 60.13	Yes	***	<0.0001

**LGPIN vs. PD**	42.69	23.18 to 62.20	Yes	***	<0.0001
**HGPIN vs. MI**	33.97	14.46 to 53.47	Yes	***	<0.0001
**HGPIN vs. WD**	21.62	2.114 to 41.13	Yes	*	0.0193
**HGPIN vs. MD**	32.00	12.49 to 51.51	Yes	***	<0.0001
**HGPIN vs. PD**	34.07	14.56 to 53.58	Yes	***	<0.0001
**MI vs. WD**	-12.34	-31.85 to 7.162	No	ns	0.4924
**MI vs. MD**	-1.966	-21.47 to 17.54	No	ns	>0.9999
**MI vs. PD**	0.1034	-19.40 to 19.61	No	ns	>0.9999
**WD vs. MD**	10.38	-9.127 to 29.89	No	ns	0.6919
**WD vs. PD**	12.45	-7.058 to 31.95	No	ns	0.4820
**MD vs. PD**	2.069	-17.44 to 21.58	No	ns	>0.9999

ns = P > 0.05; * = P ≤ 0.05; ** = P ≤ 0.01; *** = P ≤ 0.001.

Table S3. Hi Myc +/- mice.

%Area of different tumor grades → DLP: Hi-Myc$^{+/-}$ untreated control (32 weeks age)

**Number of families**	**1**				
**Number of comparisons per family**	28				
**Alpha**	0.05				
**Tukey's multiple comparisons test**	Mean Diff.	95.00% CI of diff.	Significant?	Summary	Adjusted P Value
**Normal vs. Hyperplasia**	0.000	-8.342 to 8.342	No	ns	>0.9999
**Normal vs. LGPIN**	-0.4000	-8.742 to 7.942	No	ns	>0.9999
**Normal vs. HGPIN**	-67.20	-75.54 to -58.86	Yes	***	<0.0001
**Normal vs. MIC**	-2.400	-10.74 to 5.942	No	ns	0.9874
**Normal vs. WD**	-19.20	-27.54 to -10.86	Yes	***	<0.0001
**Normal vs. MD**	-9.000	-17.34 to -0.6576	Yes	*	0.0245
**Normal vs. PD**	-1.800	-10.14 to 6.542	No	ns	0.9978
**Hyperplasia vs. LGPIN**	-0.4000	-8.742 to 7.942	No	ns	>0.9999
**Hyperplasia vs. HGPIN**	-67.20	-75.54 to -58.86	Yes	***	<0.0001
**Hyperplasia vs. MIC**	-2.400	-10.74 to 5.942	No	ns	0.9874
**Hyperplasia vs. WD**	-19.20	-27.54 to -10.86	Yes	***	<0.0001
**Hyperplasia vs. MD**	-9.000	-17.34 to -0.6576	Yes	*	0.0245
**Hyperplasia vs. PD**	-1.800	-10.14 to 6.542	No	ns	0.9978
**LGPIN vs. HGPIN**	-66.80	-75.14 to -58.46	Yes	***	<0.0001
**LGPIN vs. MIC**	-2.000	-10.34 to 6.342	No	ns	0.9958
**LGPIN vs. WD**	-18.80	-27.14 to -10.46	Yes	***	<0.0001

**LGPIN vs. MD**	-8.600	-16.94 to -0.2576	Yes	*	0.0381
**LGPIN vs. PD**	-1.400	-9.742 to 6.942	No	ns	0.9996
**HGPIN vs. MIC**	64.80	56.46 to 73.14	Yes	***	<0.0001
**HGPIN vs. WD**	48.00	39.66 to 56.34	Yes	***	<0.0001
**HGPIN vs. MD**	58.20	49.86 to 66.54	Yes	***	<0.0001
**HGPIN vs. PD**	65.40	57.06 to 73.74	Yes	***	<0.0001
**MIC vs. WD**	-16.80	-25.14 to -8.458	Yes	***	<0.0001
**MIC vs. MD**	-6.600	-14.94 to 1.742	No	ns	0.2356
**MIC vs. PD**	0.6000	-7.742 to 8.942	No	ns	>0.9999
**WD vs. MD**	10.20	1.858 to 18.54	Yes	**	0.0057
**WD vs. PD**	17.40	9.058 to 25.74	Yes	***	<0.0001
**MD vs. PD**	7.200	-1.142 to 15.54	No	ns	0.1466

%Area of different tumor grades → DLP: Hi-Myc$^{+/-}$ + ASP 700 (32 weeks age)

**Number of families**	**1**				
**Number of comparisons per family**	28				
**Alpha**	0.05				
**Tukey's multiple comparisons test**	Mean Diff.	95.00% CI of diff.	Significant?	Summary	Adjusted P Value
**Normal vs. Hyperplasia**	0.000	-10.57 to 10.57	No	ns	>0.9999
**Normal vs. LGPIN**	0.000	-10.57 to 10.57	No	ns	>0.9999
**Normal vs. HGPIN**	-68.75	-79.32 to -58.18	Yes	***	<0.0001
**Normal vs. MIC**	-5.000	-15.57 to 5.574	No	ns	0.8325
**Normal vs. WD**	-7.292	-17.87 to 3.282	No	ns	0.4097
**Normal vs. MD**	-18.96	-29.53 to -8.384	Yes	***	<0.0001
**Normal vs. PD**	0.000	-10.57 to 10.57	No	ns	>0.9999
**Hyperplasia vs. LGPIN**	0.000	-10.57 to 10.57	No	ns	>0.9999
**Hyperplasia vs. HGPIN**	-68.75	-79.32 to -58.18	Yes	***	<0.0001
**Hyperplasia vs. MIC**	-5.000	-15.57 to 5.574	No	ns	0.8325
**Hyperplasia vs. WD**	-7.292	-17.87 to 3.282	No	ns	0.4097
**Hyperplasia vs. MD**	-18.96	-29.53 to -8.384	Yes	***	<0.0001
**Hyperplasia vs. PD**	0.000	-10.57 to 10.57	No	ns	>0.9999
**LGPIN vs. HGPIN**	-68.75	-79.32 to -58.18	Yes	***	<0.0001
**LGPIN vs. MIC**	-5.000	-15.57 to 5.574	No	ns	0.8325
**LGPIN vs. WD**	-7.292	-17.87 to 3.282	No	ns	0.4097
**LGPIN vs. MD**	-18.96	-29.53 to -8.384	Yes	***	<0.0001
**LGPIN vs. PD**	0.000	-10.57 to 10.57	No	ns	>0.9999
**HGPIN vs. MIC**	63.75	53.18 to 74.32	Yes	***	<0.0001
**HGPIN vs. WD**	61.46	50.88 to 72.03	Yes	***	<0.0001

**HGPIN vs. MD**	49.79	39.22 to 60.37	Yes	***	<0.0001
**HGPIN vs. PD**	68.75	58.18 to 79.32	Yes	***	<0.0001
**MIC vs. WD**	-2.292	-12.87 to 8.282	No	ns	0.9978
**MIC vs. MD**	-13.96	-24.53 to -3.384	Yes	**	0.0019
**MIC vs. PD**	5.000	-5.574 to 15.57	No	ns	0.8325
**WD vs. MD**	-11.67	-22.24 to -1.093	Yes	*	0.0194
**WD vs. PD**	7.292	-3.282 to 17.87	No	ns	0.4097
**MD vs. PD**	18.96	8.384 to 29.53	Yes	***	<0.0001

%Area of different tumor grades → DLP: Hi-Myc$^{+/-}$ + ASP 1400 (32 weeks age)

**Number of families**	**1**				
**Number of comparisons per family**	28				
**Alpha**	0.05				
**Tukey's multiple comparisons test**	Mean Diff.	95.00% CI of diff.	Significant?	Summary	Adjusted P Value
**Normal vs. Hyperplasia**	0.000	-13.54 to 13.54	No	ns	>0.9999
**Normal vs. LGPIN**	-1.429	-14.97 to 12.11	No	ns	>0.9999
**Normal vs. HGPIN**	-56.33	-69.88 to -42.79	Yes	***	<0.0001
**Normal vs. MIC**	-2.571	-16.11 to 10.97	No	ns	0.9990
**Normal vs. WD**	-11.33	-24.88 to 2.208	No	ns	0.1746
**Normal vs. MD**	-28.33	-41.88 to -14.79	Yes	***	<0.0001
**Normal vs. PD**	0.000	-13.54 to 13.54	No	ns	>0.9999
**Hyperplasia vs. LGPIN**	-1.429	-14.97 to 12.11	No	ns	>0.9999
**Hyperplasia vs. HGPIN**	-56.33	-69.88 to -42.79	Yes	***	<0.0001
**Hyperplasia vs. MIC**	-2.571	-16.11 to 10.97	No	ns	0.9990
**Hyperplasia vs. WD**	-11.33	-24.88 to 2.208	No	ns	0.1746
**Hyperplasia vs. MD**	-28.33	-41.88 to -14.79	Yes	***	<0.0001
**Hyperplasia vs. PD**	0.000	-13.54 to 13.54	No	ns	>0.9999
**LGPIN vs. HGPIN**	-54.90	-68.45 to -41.36	Yes	***	<0.0001
**LGPIN vs. MIC**	-1.143	-14.68 to 12.40	No	ns	>0.9999
**LGPIN vs. WD**	-9.905	-23.45 to 3.637	No	ns	0.3301
**LGPIN vs. MD**	-26.90	-40.45 to -13.36	Yes	***	<0.0001
**LGPIN vs. PD**	1.429	-12.11 to 14.97	No	ns	>0.9999
**HGPIN vs. MIC**	53.76	40.22 to 67.30	Yes	***	<0.0001
**HGPIN vs. WD**	45.00	31.46 to 58.54	Yes	***	<0.0001
**HGPIN vs. MD**	28.00	14.46 to 41.54	Yes	***	<0.0001
**HGPIN vs. PD**	56.33	42.79 to 69.88	Yes	***	<0.0001
**MIC vs. WD**	-8.762	-22.30 to 4.780	No	ns	0.4937
**MIC vs. MD**	-25.76	-39.30 to -12.22	Yes	***	<0.0001

**MIC vs. PD**	2.571	-10.97 to 16.11	No	ns	0.9990
**WD vs. MD**	-17.00	-30.54 to -3.458	Yes	**	0.0041
**WD vs. PD**	11.33	-2.208 to 24.88	No	ns	0.1746
**MD vs. PD**	28.33	14.79 to 41.88	Yes	***	<0.0001

%Area of different tumor grades → DLP: Hi-Myc$^{+/-}$ + NAP 200 (32 weeks age)

**Number of families**	**1**				
**Number of comparisons per family**	28				
**Alpha**	0.05				
**Tukey's multiple comparisons test**	Mean Diff.	95.00% CI of diff.	Significant?	Summary	Adjusted P Value
**Normal vs. Hyperplasia**	0.000	-10.40 to 10.40	No	ns	>0.9999
**Normal vs. LGPIN**	-5.952	-16.35 to 4.446	No	ns	0.6490
**Normal vs. HGPIN**	-71.90	-82.30 to -61.51	Yes	***	<0.0001
**Normal vs. MIC**	-2.476	-12.87 to 7.922	No	ns	0.9959
**Normal vs. WD**	-10.71	-21.11 to -0.3159	Yes	*	0.0383
**Normal vs. MD**	-3.952	-14.35 to 6.446	No	ns	0.9399
**Normal vs. PD**	-5.000	-15.40 to 5.398	No	ns	0.8186
**Hyperplasia vs. LGPIN**	-5.952	-16.35 to 4.446	No	ns	0.6490
**Hyperplasia vs. HGPIN**	-71.90	-82.30 to -61.51	Yes	***	<0.0001
**Hyperplasia vs. MIC**	-2.476	-12.87 to 7.922	No	ns	0.9959
**Hyperplasia vs. WD**	-10.71	-21.11 to -0.3159	Yes	*	0.0383
**Hyperplasia vs. MD**	-3.952	-14.35 to 6.446	No	ns	0.9399
**Hyperplasia vs. PD**	-5.000	-15.40 to 5.398	No	ns	0.8186
**LGPIN vs. HGPIN**	-65.95	-76.35 to -55.55	Yes	***	<0.0001
**LGPIN vs. MIC**	3.476	-6.922 to 13.87	No	ns	0.9697
**LGPIN vs. WD**	-4.762	-15.16 to 5.636	No	ns	0.8531
**LGPIN vs. MD**	2.000	-8.398 to 12.40	No	ns	0.9990
**LGPIN vs. PD**	0.9524	-9.446 to 11.35	No	ns	>0.9999
**HGPIN vs. MIC**	69.43	59.03 to 79.83	Yes	***	<0.0001
**HGPIN vs. WD**	61.19	50.79 to 71.59	Yes	***	<0.0001
**HGPIN vs. MD**	67.95	57.55 to 78.35	Yes	***	<0.0001
**HGPIN vs. PD**	66.90	56.51 to 77.30	Yes	***	<0.0001
**MIC vs. WD**	-8.238	-18.64 to 2.160	No	ns	0.2327
**MIC vs. MD**	-1.476	-11.87 to 8.922	No	ns	0.9999
**MIC vs. PD**	-2.524	-12.92 to 7.875	No	ns	0.9954
**WD vs. MD**	6.762	-3.636 to 17.16	No	ns	0.4870
**WD vs. PD**	5.714	-4.684 to 16.11	No	ns	0.6951
**MD vs. PD**	-1.048	-11.45 to 9.351	No	ns	>0.9999

%Area of different tumor grades → DLP: Hi-Myc$^{+/-}$ + NAP 400 (32 weeks age)

**Number of families**	**1**				
**Number of comparisons per family**	28				
**Alpha**	0.05				
**Tukey's multiple comparisons test**	Mean Diff.	95.00% CI of diff.	Significant?	Summary	Adjusted P Value
**Normal vs. Hyperplasia**	0.000	-10.69 to 10.69	No	ns	>0.9999
**Normal vs. LGPIN**	-12.40	-23.09 to -1.712	Yes	*	0.0110
**Normal vs. HGPIN**	-69.20	-79.89 to -58.51	Yes	***	<0.0001
**Normal vs. MIC**	-4.120	-14.81 to 6.568	No	ns	0.9364
**Normal vs. WD**	-10.08	-20.77 to 0.6084	No	ns	0.0803
**Normal vs. MD**	-4.200	-14.89 to 6.488	No	ns	0.9299
**Normal vs. PD**	0.000	-10.69 to 10.69	No	ns	>0.9999
**Hyperplasia vs. LGPIN**	-12.40	-23.09 to -1.712	Yes	*	0.0110
**Hyperplasia vs. HGPIN**	-69.20	-79.89 to -58.51	Yes	***	<0.0001
**Hyperplasia vs. MIC**	-4.120	-14.81 to 6.568	No	ns	0.9364
**Hyperplasia vs. WD**	-10.08	-20.77 to 0.6084	No	ns	0.0803
**Hyperplasia vs. MD**	-4.200	-14.89 to 6.488	No	ns	0.9299
**Hyperplasia vs. PD**	0.000	-10.69 to 10.69	No	ns	>0.9999
**LGPIN vs. HGPIN**	-56.80	-67.49 to -46.11	Yes	***	<0.0001
**LGPIN vs. MIC**	8.280	-2.408 to 18.97	No	ns	0.2602
**LGPIN vs. WD**	2.320	-8.368 to 13.01	No	ns	0.9978
**LGPIN vs. MD**	8.200	-2.488 to 18.89	No	ns	0.2718
**LGPIN vs. PD**	12.40	1.712 to 23.09	Yes	*	0.0110
**HGPIN vs. MIC**	65.08	54.39 to 75.77	Yes	***	<0.0001
**HGPIN vs. WD**	59.12	48.43 to 69.81	Yes	***	<0.0001
**HGPIN vs. MD**	65.00	54.31 to 75.69	Yes	***	<0.0001
**HGPIN vs. PD**	69.20	58.51 to 79.89	Yes	***	<0.0001
**MIC vs. WD**	-5.960	-16.65 to 4.728	No	ns	0.6815
**MIC vs. MD**	-0.08000	-10.77 to 10.61	No	ns	>0.9999
**MIC vs. PD**	4.120	-6.568 to 14.81	No	ns	0.9364
**WD vs. MD**	5.880	-4.808 to 16.57	No	ns	0.6963
**WD vs. PD**	10.08	-0.6084 to 20.77	No	ns	0.0803
**MD vs. PD**	4.200	-6.488 to 14.89	No	ns	0.9299

%Area of different tumor grades → VP: Hi-Myc$^{+/-}$ untreated control (32 weeks age)

Number of families	1				
**Number of comparisons per family**	28				
**Alpha**	0.05				
**Tukey's multiple comparisons test**	Mean Diff.	95.00% CI of diff.	Significant?	Summary	Adjusted P Value
**Normal vs. Hyperplasia**	0.000	-12.39 to 12.39	No	ns	>0.9999
**Normal vs. LGPIN**	-20.43	-32.82 to -8.045	Yes	***	<0.0001
**Normal vs. HGPIN**	-76.57	-88.95 to -64.18	Yes	***	<0.0001
**Normal vs. MIC**	-2.348	-14.74 to 10.04	No	ns	0.9991
**Normal vs. WD**	-0.6522	-13.04 to 11.74	No	ns	>0.9999
**Normal vs. MD**	0.000	-12.39 to 12.39	No	ns	>0.9999
**Normal vs. PD**	0.000	-12.39 to 12.39	No	ns	>0.9999
**Hyperplasia vs. LGPIN**	-20.43	-32.82 to -8.045	Yes	***	<0.0001
**Hyperplasia vs. HGPIN**	-76.57	-88.95 to -64.18	Yes	***	<0.0001
**Hyperplasia vs. MIC**	-2.348	-14.74 to 10.04	No	ns	0.9991
**Hyperplasia vs. WD**	-0.6522	-13.04 to 11.74	No	ns	>0.9999
**Hyperplasia vs. MD**	0.000	-12.39 to 12.39	No	ns	>0.9999
**Hyperplasia vs. PD**	0.000	-12.39 to 12.39	No	ns	>0.9999
**LGPIN vs. HGPIN**	-56.13	-68.52 to -43.74	Yes	***	<0.0001
**LGPIN vs. MIC**	18.09	5.697 to 30.48	Yes	***	0.0004
**LGPIN vs. WD**	19.78	7.393 to 32.17	Yes	***	<0.0001
**LGPIN vs. MD**	20.43	8.045 to 32.82	Yes	***	<0.0001
**LGPIN vs. PD**	20.43	8.045 to 32.82	Yes	***	<0.0001
**HGPIN vs. MIC**	74.22	61.83 to 86.61	Yes	***	<0.0001
**HGPIN vs. WD**	75.91	63.52 to 88.30	Yes	***	<0.0001
**HGPIN vs. MD**	76.57	64.18 to 88.95	Yes	***	<0.0001
**HGPIN vs. PD**	76.57	64.18 to 88.95	Yes	***	<0.0001
**MIC vs. WD**	1.696	-10.69 to 14.09	No	ns	0.9999
**MIC vs. MD**	2.348	-10.04 to 14.74	No	ns	0.9991
**MIC vs. PD**	2.348	-10.04 to 14.74	No	ns	0.9991
**WD vs. MD**	0.6522	-11.74 to 13.04	No	ns	>0.9999
**WD vs. PD**	0.6522	-11.74 to 13.04	No	ns	>0.9999
**MD vs. PD**	0.000	-12.39 to 12.39	No	ns	>0.9999

%Area of different tumor grades → VP: Hi-Myc$^{+/-}$ + ASP 700 (32 weeks age)

**Number of families**	**1**				
**Number of comparisons per family**	28				
**Alpha**	0.05				
**Tukey's multiple comparisons test**	Mean Diff.	95.00% CI of diff.	Significant?	Summary	Adjusted P Value
**Normal vs. Hyperplasia**	-1.250	-14.31 to 11.81	No	ns	>0.9999
**Normal vs. LGPIN**	-53.46	-66.52 to -40.39	Yes	***	<0.0001
**Normal vs. HGPIN**	-39.71	-52.77 to -26.64	Yes	***	<0.0001
**Normal vs. MIC**	-3.917	-16.98 to 9.147	No	ns	0.9839
**Normal vs. WD**	-1.667	-14.73 to 11.40	No	ns	>0.9999
**Normal vs. MD**	0.000	-13.06 to 13.06	No	ns	>0.9999
**Normal vs. PD**	0.000	-13.06 to 13.06	No	ns	>0.9999
**Hyperplasia vs. LGPIN**	-52.21	-65.27 to -39.14	Yes	***	<0.0001
**Hyperplasia vs. HGPIN**	-38.46	-51.52 to -25.39	Yes	***	<0.0001
**Hyperplasia vs. MIC**	-2.667	-15.73 to 10.40	No	ns	0.9985
**Hyperplasia vs. WD**	-0.4167	-13.48 to 12.65	No	ns	>0.9999
**Hyperplasia vs. MD**	1.250	-11.81 to 14.31	No	ns	>0.9999
**Hyperplasia vs. PD**	1.250	-11.81 to 14.31	No	ns	>0.9999
**LGPIN vs. HGPIN**	13.75	0.6858 to 26.81	Yes	*	0.0313
**LGPIN vs. MIC**	49.54	36.48 to 62.61	Yes	***	<0.0001
**LGPIN vs. WD**	51.79	38.73 to 64.86	Yes	***	<0.0001
**LGPIN vs. MD**	53.46	40.39 to 66.52	Yes	***	<0.0001
**LGPIN vs. PD**	53.46	40.39 to 66.52	Yes	***	<0.0001
**HGPIN vs. MIC**	35.79	22.73 to 48.86	Yes	***	<0.0001
**HGPIN vs. WD**	38.04	24.98 to 51.11	Yes	***	<0.0001
**HGPIN vs. MD**	39.71	26.64 to 52.77	Yes	***	<0.0001
**HGPIN vs. PD**	39.71	26.64 to 52.77	Yes	***	<0.0001
**MIC vs. WD**	2.250	-10.81 to 15.31	No	ns	0.9995
**MIC vs. MD**	3.917	-9.147 to 16.98	No	ns	0.9839
**MIC vs. PD**	3.917	-9.147 to 16.98	No	ns	0.9839
**WD vs. MD**	1.667	-11.40 to 14.73	No	ns	>0.9999
**WD vs. PD**	1.667	-11.40 to 14.73	No	ns	>0.9999
**MD vs. PD**	0.000	-13.06 to 13.06	No	ns	>0.9999

%Area of different tumor grades → VP: Hi-Myc$^{+/-}$ + ASP 1400 (32 weeks age)

Number of families	1				
Number of comparisons per family	28				
Alpha	0.05				
Tukey's multiple comparisons test	Mean Diff.	95.00% CI of diff.	Significant?	Summary	Adjusted P Value
Normal vs. Hyperplasia	-8.125	-21.47 to 5.218	No	ns	0.5752
Normal vs. LGPIN	-51.17	-64.51 to -37.82	Yes	***	<0.0001
Normal vs. HGPIN	-33.42	-46.76 to -20.07	Yes	***	<0.0001
Normal vs. MIC	-1.875	-15.22 to 11.47	No	ns	0.9999
Normal vs. WD	-2.708	-16.05 to 10.63	No	ns	0.9985
Normal vs. MD	-2.708	-16.05 to 10.63	No	ns	0.9985
Normal vs. PD	0.000	-13.34 to 13.34	No	ns	>0.9999
Hyperplasia vs. LGPIN	-43.04	-56.38 to -29.70	Yes	***	<0.0001
Hyperplasia vs. HGPIN	-25.29	-38.63 to -11.95	Yes	***	<0.0001
Hyperplasia vs. MIC	6.250	-7.093 to 19.59	No	ns	0.8392
Hyperplasia vs. WD	5.417	-7.926 to 18.76	No	ns	0.9173
Hyperplasia vs. MD	5.417	-7.926 to 18.76	No	ns	0.9173
Hyperplasia vs. PD	8.125	-5.218 to 21.47	No	ns	0.5752
LGPIN vs. HGPIN	17.75	4.407 to 31.09	Yes	**	0.0017
LGPIN vs. MIC	49.29	35.95 to 62.63	Yes	***	<0.0001
LGPIN vs. WD	48.46	35.12 to 61.80	Yes	***	<0.0001
LGPIN vs. MD	48.46	35.12 to 61.80	Yes	***	<0.0001
LGPIN vs. PD	51.17	37.82 to 64.51	Yes	***	<0.0001
HGPIN vs. MIC	31.54	18.20 to 44.88	Yes	***	<0.0001
HGPIN vs. WD	30.71	17.37 to 44.05	Yes	***	<0.0001
HGPIN vs. MD	30.71	17.37 to 44.05	Yes	***	<0.0001
HGPIN vs. PD	33.42	20.07 to 46.76	Yes	***	<0.0001
MIC vs. WD	-0.8333	-14.18 to 12.51	No	ns	>0.9999
MIC vs. MD	-0.8333	-14.18 to 12.51	No	ns	>0.9999
MIC vs. PD	1.875	-11.47 to 15.22	No	ns	0.9999
WD vs. MD	0.000	-13.34 to 13.34	No	ns	>0.9999
WD vs. PD	2.708	-10.63 to 16.05	No	ns	0.9985
MD vs. PD	2.708	-10.63 to 16.05	No	ns	0.9985

%Area of different tumor grades → VP: Hi-Myc$^{+/-}$ + NAP 200 (32 weeks age)

**Number of families**	**1**				
**Number of comparisons per family**	28				
**Alpha**	0.05				
**Tukey's multiple comparisons test**	Mean Diff.	95.00% CI of diff.	Significant?	Summary	Adjusted P Value
**Normal vs. Hyperplasia**	0.000	-15.43 to 15.43	No	ns	>0.9999
**Normal vs. LGPIN**	-33.42	-48.86 to -17.99	Yes	***	<0.0001
**Normal vs. HGPIN**	-63.16	-78.59 to -47.72	Yes	***	<0.0001
**Normal vs. MIC**	-2.632	-18.07 to 12.80	No	ns	0.9995
**Normal vs. WD**	-0.7895	-16.22 to 14.65	No	ns	>0.9999
**Normal vs. MD**	0.000	-15.43 to 15.43	No	ns	>0.9999
**Normal vs. PD**	0.000	-15.43 to 15.43	No	ns	>0.9999
**Hyperplasia vs. LGPIN**	-33.42	-48.86 to -17.99	Yes	***	<0.0001
**Hyperplasia vs. HGPIN**	-63.16	-78.59 to -47.72	Yes	***	<0.0001
**Hyperplasia vs. MIC**	-2.632	-18.07 to 12.80	No	ns	0.9995
**Hyperplasia vs. WD**	-0.7895	-16.22 to 14.65	No	ns	>0.9999
**Hyperplasia vs. MD**	0.000	-15.43 to 15.43	No	ns	>0.9999
**Hyperplasia vs. PD**	0.000	-15.43 to 15.43	No	ns	>0.9999
**LGPIN vs. HGPIN**	-29.74	-45.17 to -14.30	Yes	***	<0.0001
**LGPIN vs. MIC**	30.79	15.35 to 46.22	Yes	***	<0.0001
**LGPIN vs. WD**	32.63	17.20 to 48.07	Yes	***	<0.0001
**LGPIN vs. MD**	33.42	17.99 to 48.86	Yes	***	<0.0001
**LGPIN vs. PD**	33.42	17.99 to 48.86	Yes	***	<0.0001
**HGPIN vs. MIC**	60.53	45.09 to 75.96	Yes	***	<0.0001
**HGPIN vs. WD**	62.37	46.93 to 77.80	Yes	***	<0.0001
**HGPIN vs. MD**	63.16	47.72 to 78.59	Yes	***	<0.0001
**HGPIN vs. PD**	63.16	47.72 to 78.59	Yes	***	<0.0001
**MIC vs. WD**	1.842	-13.59 to 17.28	No	ns	>0.9999
**MIC vs. MD**	2.632	-12.80 to 18.07	No	ns	0.9995
**MIC vs. PD**	2.632	-12.80 to 18.07	No	ns	0.9995
**WD vs. MD**	0.7895	-14.65 to 16.22	No	ns	>0.9999
**WD vs. PD**	0.7895	-14.65 to 16.22	No	ns	>0.9999
**MD vs. PD**	0.000	-15.43 to 15.43	No	ns	>0.9999

%Area of different tumor grades → VP: Hi-Myc$^{+/-}$ + NAP 400 (32 weeks age)

Number of families	1				
Number of comparisons per family	28				
Alpha	0.05				
Tukey's multiple comparisons test	Mean Diff.	95.00% CI of diff.	Significant?	Summary	Adjusted P Value
Normal vs. Hyperplasia	-0.9091	-14.37 to 12.55	No	ns	>0.9999
Normal vs. LGPIN	-41.59	-55.05 to -28.13	Yes	***	<0.0001
Normal vs. HGPIN	-55.14	-68.59 to -41.68	Yes	***	<0.0001
Normal vs. MIC	-2.364	-15.82 to 11.09	No	ns	0.9994
Normal vs. WD	0.000	-13.46 to 13.46	No	ns	>0.9999
Normal vs. MD	0.000	-13.46 to 13.46	No	ns	>0.9999
Normal vs. PD	0.000	-13.46 to 13.46	No	ns	>0.9999
Hyperplasia vs. LGPIN	-40.68	-54.14 to -27.22	Yes	***	<0.0001
Hyperplasia vs. HGPIN	-54.23	-67.68 to -40.77	Yes	***	<0.0001
Hyperplasia vs. MIC	-1.455	-14.91 to 12.00	No	ns	>0.9999
Hyperplasia vs. WD	0.9091	-12.55 to 14.37	No	ns	>0.9999
Hyperplasia vs. MD	0.9091	-12.55 to 14.37	No	ns	>0.9999
Hyperplasia vs. PD	0.9091	-12.55 to 14.37	No	ns	>0.9999
LGPIN vs. HGPIN	-13.55	-27.00 to -0.08805	Yes	*	0.0473
LGPIN vs. MIC	39.23	25.77 to 52.68	Yes	***	<0.0001
LGPIN vs. WD	41.59	28.13 to 55.05	Yes	***	<0.0001
LGPIN vs. MD	41.59	28.13 to 55.05	Yes	***	<0.0001
LGPIN vs. PD	41.59	28.13 to 55.05	Yes	***	<0.0001
HGPIN vs. MIC	52.77	39.32 to 66.23	Yes	***	<0.0001
HGPIN vs. WD	55.14	41.68 to 68.59	Yes	***	<0.0001
HGPIN vs. MD	55.14	41.68 to 68.59	Yes	***	<0.0001
HGPIN vs. PD	55.14	41.68 to 68.59	Yes	***	<0.0001
MIC vs. WD	2.364	-11.09 to 15.82	No	ns	0.9994
MIC vs. MD	2.364	-11.09 to 15.82	No	ns	0.9994
MIC vs. PD	2.364	-11.09 to 15.82	No	ns	0.9994
WD vs. MD	0.000	-13.46 to 13.46	No	ns	>0.9999
WD vs. PD	0.000	-13.46 to 13.46	No	ns	>0.9999
MD vs. PD	0.000	-13.46 to 13.46	No	ns	>0.9999

%Area of different tumor grades → AP: Hi-Myc$^{+/-}$ untreated control (32 weeks age)

**Number of families**	**1**				
**Number of comparisons per family**	28				
**Alpha**	0.05				
**Tukey's multiple comparisons test**	Mean Diff.	95.00% CI of diff.	Significant?	Summary	Adjusted P Value
**Normal vs. Hyperplasia**	-3.846	-20.42 to 12.73	No	ns	0.9966
**Normal vs. LGPIN**	-56.92	-73.50 to -40.34	Yes	***	<0.0001
**Normal vs. HGPIN**	-39.23	-55.81 to -22.65	Yes	***	<0.0001
**Normal vs. MIC**	0.000	-16.58 to 16.58	No	ns	>0.9999
**Normal vs. WD**	0.000	-16.58 to 16.58	No	ns	>0.9999
**Normal vs. MD**	0.000	-16.58 to 16.58	No	ns	>0.9999
**Normal vs. PD**	0.000	-16.58 to 16.58	No	ns	>0.9999
**Hyperplasia vs. LGPIN**	-53.08	-69.66 to -36.50	Yes	***	<0.0001
**Hyperplasia vs. HGPIN**	-35.38	-51.96 to -18.81	Yes	***	<0.0001
**Hyperplasia vs. MIC**	3.846	-12.73 to 20.42	No	ns	0.9966
**Hyperplasia vs. WD**	3.846	-12.73 to 20.42	No	ns	0.9966
**Hyperplasia vs. MD**	3.846	-12.73 to 20.42	No	ns	0.9966
**Hyperplasia vs. PD**	3.846	-12.73 to 20.42	No	ns	0.9966
**LGPIN vs. HGPIN**	17.69	1.114 to 34.27	Yes	*	0.0273
**LGPIN vs. MIC**	56.92	40.34 to 73.50	Yes	***	<0.0001
**LGPIN vs. WD**	56.92	40.34 to 73.50	Yes	***	<0.0001
**LGPIN vs. MD**	56.92	40.34 to 73.50	Yes	***	<0.0001
**LGPIN vs. PD**	56.92	40.34 to 73.50	Yes	***	<0.0001
**HGPIN vs. MIC**	39.23	22.65 to 55.81	Yes	***	<0.0001
**HGPIN vs. WD**	39.23	22.65 to 55.81	Yes	***	<0.0001
**HGPIN vs. MD**	39.23	22.65 to 55.81	Yes	***	<0.0001
**HGPIN vs. PD**	39.23	22.65 to 55.81	Yes	***	<0.0001
**MIC vs. WD**	0.000	-16.58 to 16.58	No	ns	>0.9999
**MIC vs. MD**	0.000	-16.58 to 16.58	No	ns	>0.9999
**MIC vs. PD**	0.000	-16.58 to 16.58	No	ns	>0.9999
**WD vs. MD**	0.000	-16.58 to 16.58	No	ns	>0.9999
**WD vs. PD**	0.000	-16.58 to 16.58	No	ns	>0.9999
**MD vs. PD**	0.000	-16.58 to 16.58	No	ns	>0.9999

%Area of different tumor grades → AP: Hi-Myc$^{+/-}$ + ASP 700 (32 weeks age)

**Number of families**	**1**				
**Number of comparisons per family**	28				
**Alpha**	0.05				
**Tukey's multiple comparisons test**	Mean Diff.	95.00% CI of diff.	Significant?	Summary	Adjusted P Value
**Normal vs. Hyperplasia**	-25.60	-40.82 to -10.38	Yes	***	<0.0001
**Normal vs. LGPIN**	-46.80	-62.02 to -31.58	Yes	***	<0.0001
**Normal vs. HGPIN**	-27.60	-42.82 to -12.38	Yes	***	<0.0001
**Normal vs. MIC**	0.000	-15.22 to 15.22	No	ns	>0.9999
**Normal vs. WD**	0.000	-15.22 to 15.22	No	ns	>0.9999
**Normal vs. MD**	0.000	-15.22 to 15.22	No	ns	>0.9999
**Normal vs. PD**	0.000	-15.22 to 15.22	No	ns	>0.9999
**Hyperplasia vs. LGPIN**	-21.20	-36.42 to -5.984	Yes	***	0.0008
**Hyperplasia vs. HGPIN**	-2.000	-17.22 to 13.22	No	ns	>0.9999
**Hyperplasia vs. MIC**	25.60	10.38 to 40.82	Yes	***	<0.0001
**Hyperplasia vs. WD**	25.60	10.38 to 40.82	Yes	***	<0.0001
**Hyperplasia vs. MD**	25.60	10.38 to 40.82	Yes	***	<0.0001
**Hyperplasia vs. PD**	25.60	10.38 to 40.82	Yes	***	<0.0001
**LGPIN vs. HGPIN**	19.20	3.984 to 34.42	Yes	**	0.0037
**LGPIN vs. MIC**	46.80	31.58 to 62.02	Yes	***	<0.0001
**LGPIN vs. WD**	46.80	31.58 to 62.02	Yes	***	<0.0001
**LGPIN vs. MD**	46.80	31.58 to 62.02	Yes	***	<0.0001
**LGPIN vs. PD**	46.80	31.58 to 62.02	Yes	***	<0.0001
**HGPIN vs. MIC**	27.60	12.38 to 42.82	Yes	***	<0.0001
**HGPIN vs. WD**	27.60	12.38 to 42.82	Yes	***	<0.0001
**HGPIN vs. MD**	27.60	12.38 to 42.82	Yes	***	<0.0001
**HGPIN vs. PD**	27.60	12.38 to 42.82	Yes	***	<0.0001
**MIC vs. WD**	0.000	-15.22 to 15.22	No	ns	>0.9999
**MIC vs. MD**	0.000	-15.22 to 15.22	No	ns	>0.9999
**MIC vs. PD**	0.000	-15.22 to 15.22	No	ns	>0.9999
**WD vs. MD**	0.000	-15.22 to 15.22	No	ns	>0.9999
**WD vs. PD**	0.000	-15.22 to 15.22	No	ns	>0.9999
**MD vs. PD**	0.000	-15.22 to 15.22	No	ns	>0.9999

%Area of different tumor grades → AP: Hi-Myc$^{+/-}$ + ASP 1400 (32 weeks age)

Number of families	1				
Number of comparisons per family	28				
Alpha	0.05				
Tukey's multiple comparisons test	Mean Diff.	95.00% CI of diff.	Significant?	Summary	Adjusted P Value
Normal vs. Hyperplasia	-38.20	-52.06 to -24.34	Yes	***	<0.0001
Normal vs. LGPIN	-43.60	-57.46 to -29.74	Yes	***	<0.0001
Normal vs. HGPIN	-17.00	-30.86 to -3.138	Yes	**	0.0055
Normal vs. MIC	-0.8000	-14.66 to 13.06	No	ns	>0.9999
Normal vs. WD	-0.4000	-14.26 to 13.46	No	ns	>0.9999
Normal vs. MD	0.000	-13.86 to 13.86	No	ns	>0.9999
Normal vs. PD	0.000	-13.86 to 13.86	No	ns	>0.9999
Hyperplasia vs. LGPIN	-5.400	-19.26 to 8.462	No	ns	0.9329
Hyperplasia vs. HGPIN	21.20	7.338 to 35.06	Yes	***	0.0001
Hyperplasia vs. MIC	37.40	23.54 to 51.26	Yes	***	<0.0001
Hyperplasia vs. WD	37.80	23.94 to 51.66	Yes	***	<0.0001
Hyperplasia vs. MD	38.20	24.34 to 52.06	Yes	***	<0.0001
Hyperplasia vs. PD	38.20	24.34 to 52.06	Yes	***	<0.0001
LGPIN vs. HGPIN	26.60	12.74 to 40.46	Yes	***	<0.0001
LGPIN vs. MIC	42.80	28.94 to 56.66	Yes	***	<0.0001
LGPIN vs. WD	43.20	29.34 to 57.06	Yes	***	<0.0001
LGPIN vs. MD	43.60	29.74 to 57.46	Yes	***	<0.0001
LGPIN vs. PD	43.60	29.74 to 57.46	Yes	***	<0.0001
HGPIN vs. MIC	16.20	2.338 to 30.06	Yes	*	0.0101
HGPIN vs. WD	16.60	2.738 to 30.46	Yes	**	0.0074
HGPIN vs. MD	17.00	3.138 to 30.86	Yes	**	0.0055
HGPIN vs. PD	17.00	3.138 to 30.86	Yes	**	0.0055
MIC vs. WD	0.4000	-13.46 to 14.26	No	ns	>0.9999
MIC vs. MD	0.8000	-13.06 to 14.66	No	ns	>0.9999
MIC vs. PD	0.8000	-13.06 to 14.66	No	ns	>0.9999
WD vs. MD	0.4000	-13.46 to 14.26	No	ns	>0.9999
WD vs. PD	0.4000	-13.46 to 14.26	No	ns	>0.9999
MD vs. PD	0.000	-13.86 to 13.86	No	ns	>0.9999

%Area of different tumor grades → AP: Hi-Myc$^{+/-}$ + NAP 200 (32 weeks age)

Number of families	1				
Number of comparisons per family	28				
Alpha	0.05				

**Tukey's multiple comparisons test**	Mean Diff.	95.00% CI of diff.	Significant?	Summary	Adjusted P Value
**Normal vs. Hyperplasia**	-15.00	-30.48 to 0.4804	No	ns	0.0650
**Normal vs. LGPIN**	-57.40	-72.88 to -41.92	Yes	***	<0.0001
**Normal vs. HGPIN**	-27.40	-42.88 to -11.92	Yes	***	<0.0001
**Normal vs. MIC**	-0.2000	-15.68 to 15.28	No	ns	>0.9999
**Normal vs. WD**	0.000	-15.48 to 15.48	No	ns	>0.9999
**Normal vs. MD**	0.000	-15.48 to 15.48	No	ns	>0.9999
**Normal vs. PD**	0.000	-15.48 to 15.48	No	ns	>0.9999
**Hyperplasia vs. LGPIN**	-42.40	-57.88 to -26.92	Yes	***	<0.0001
**Hyperplasia vs. HGPIN**	-12.40	-27.88 to 3.080	No	ns	0.2216
**Hyperplasia vs. MIC**	14.80	-0.6804 to 30.28	No	ns	0.0723
**Hyperplasia vs. WD**	15.00	-0.4804 to 30.48	No	ns	0.0650
**Hyperplasia vs. MD**	15.00	-0.4804 to 30.48	No	ns	0.0650
**Hyperplasia vs. PD**	15.00	-0.4804 to 30.48	No	ns	0.0650
**LGPIN vs. HGPIN**	30.00	14.52 to 45.48	Yes	***	<0.0001
**LGPIN vs. MIC**	57.20	41.72 to 72.68	Yes	***	<0.0001
**LGPIN vs. WD**	57.40	41.92 to 72.88	Yes	***	<0.0001
**LGPIN vs. MD**	57.40	41.92 to 72.88	Yes	***	<0.0001
**LGPIN vs. PD**	57.40	41.92 to 72.88	Yes	***	<0.0001
**HGPIN vs. MIC**	27.20	11.72 to 42.68	Yes	***	<0.0001
**HGPIN vs. WD**	27.40	11.92 to 42.88	Yes	***	<0.0001
**HGPIN vs. MD**	27.40	11.92 to 42.88	Yes	***	<0.0001
**HGPIN vs. PD**	27.40	11.92 to 42.88	Yes	***	<0.0001
**MIC vs. WD**	0.2000	-15.28 to 15.68	No	ns	>0.9999
**MIC vs. MD**	0.2000	-15.28 to 15.68	No	ns	>0.9999
**MIC vs. PD**	0.2000	-15.28 to 15.68	No	ns	>0.9999
**WD vs. MD**	0.000	-15.48 to 15.48	No	ns	>0.9999
**WD vs. PD**	0.000	-15.48 to 15.48	No	ns	>0.9999
**MD vs. PD**	0.000	-15.48 to 15.48	No	ns	>0.9999

%Area of different tumor grades → AP: Hi-Myc$^{+/-}$ + NAP 400 (32 weeks age)

**Number of families**	**1**				
**Number of comparisons per family**	28				
**Alpha**	0.05				
**Tukey's multiple comparisons test**	Mean Diff.	95.00% CI of diff.	Significant?	Summary	Adjusted P Value
**Normal vs. Hyperplasia**	-19.20	-33.54 to -4.860	Yes	**	0.0015
**Normal vs. LGPIN**	-44.60	-58.94 to -30.26	Yes	***	<0.0001
**Normal vs. HGPIN**	-36.20	-50.54 to -21.86	Yes	***	<0.0001

**Normal vs. MIC**	0.000	-14.34 to 14.34	No	ns	>0.9999
**Normal vs. WD**	0.000	-14.34 to 14.34	No	ns	>0.9999
**Normal vs. MD**	0.000	-14.34 to 14.34	No	ns	>0.9999
**Normal vs. PD**	0.000	-14.34 to 14.34	No	ns	>0.9999
**Hyperplasia vs. LGPIN**	-25.40	-39.74 to -11.06	Yes	***	<0.0001
**Hyperplasia vs. HGPIN**	-17.00	-31.34 to -2.660	Yes	**	0.0085
**Hyperplasia vs. MIC**	19.20	4.860 to 33.54	Yes	**	0.0015
**Hyperplasia vs. WD**	19.20	4.860 to 33.54	Yes	**	0.0015
**Hyperplasia vs. MD**	19.20	4.860 to 33.54	Yes	**	0.0015
**Hyperplasia vs. PD**	19.20	4.860 to 33.54	Yes	**	0.0015
**LGPIN vs. HGPIN**	8.400	-5.940 to 22.74	No	ns	0.6241
**LGPIN vs. MIC**	44.60	30.26 to 58.94	Yes	***	<0.0001
**LGPIN vs. WD**	44.60	30.26 to 58.94	Yes	***	<0.0001
**LGPIN vs. MD**	44.60	30.26 to 58.94	Yes	***	<0.0001
**LGPIN vs. PD**	44.60	30.26 to 58.94	Yes	***	<0.0001
**HGPIN vs. MIC**	36.20	21.86 to 50.54	Yes	***	<0.0001
**HGPIN vs. WD**	36.20	21.86 to 50.54	Yes	***	<0.0001
**HGPIN vs. MD**	36.20	21.86 to 50.54	Yes	***	<0.0001
**HGPIN vs. PD**	36.20	21.86 to 50.54	Yes	***	<0.0001
**MIC vs. WD**	0.000	-14.34 to 14.34	No	ns	>0.9999
**MIC vs. MD**	0.000	-14.34 to 14.34	No	ns	>0.9999
**MIC vs. PD**	0.000	-14.34 to 14.34	No	ns	>0.9999
**WD vs. MD**	0.000	-14.34 to 14.34	No	ns	>0.9999
**WD vs. PD**	0.000	-14.34 to 14.34	No	ns	>0.9999
**MD vs. PD**	0.000	-14.34 to 14.34	No	ns	>0.9999
